# Exploration of the structural diversity and distribution pattern of gangliosides and sulfatides in mouse brain tissues and biomarkers for Parkinson’s Disease

**DOI:** 10.1186/s12944-026-02918-1

**Published:** 2026-03-25

**Authors:** Ming-yang Liu, Juan Wang, Xiong-yu Meng, Jia-ru Liu, Xiao-jun Yao, Yu-wei Wang, Lee-fong Yau, An-ran Sun, Jia-ning Mi, Liang Liu, Wen-Jing Ren, Yue Kang, Jing-rong Wang

**Affiliations:** 1 The Second Clinical College, Guangzhou University of Chinese Medicine, Chinese Medicine Guangdong Laboratory, Guangzhou, China; 2 State Key Laboratory of Traditional Chinese Medicine Syndrome, Guangzhou, China; 3 School of Pharmaceutical Sciences, Zhejiang Chinese Medical University, Hangzhou, China; 4 Centre for Artificial Intelligence Driven Drug Discovery, Faculty of Applied Sciences, Macao Polytechnic University, Macao, China; 5 College of Pharmacy, Shaanxi University of Chinese Medicine, Xianyang, China; 6 Macau University of Science and Technology, Macau, China; 7 College of Pharmacy, Nanjing University of Chinese Medicine, Nanjing, China

**Keywords:** Acidic glycosphingolipids, Gangliosides, Sulfoglycosphingolipids, Parkinson Disease, UHPLC-Q-TOF MS

## Abstract

**Background:**

Acidic glycosphingolipids (GSLs), including gangliosides (GAs) and sulfatides (STs), play key roles in intercellular signalling and neurodegenerative diseases. However, their comprehensive characterization remains challenging because of structural complexity, abundance variation, and interference from isomers and isotopes. This study aimed to systematically profile GAs and STs in mouse brain tissues and explore their potential as biomarkers in a Parkinson’s disease (PD) model.

**Methods:**

Prefractionation in combination with UHPLC-Q-TOF MS was employed for high-resolution MS and MS/MS analysis of acidic GSLs. Quantitative structure–retention relationship (QSRR) models were constructed from identified structures and retention times (RTs) to predict RTs and provide complementary support for MS/MS-based annotation. Dynamic multiple reaction monitoring (dMRM) on UHPLC-QQQ MS was applied to quantify GSLs, followed by a comparative analysis of PD and control mouse brain tissues.

**Results:**

A total of 220 acidic GSLs were characterized, including 173 GAs (21 subclasses) and 47 STs (3 subclasses), representing the most extensive profile reported from mouse brain tissues, among which 75 GAs and 29 STs are, to the best of the authors’ knowledge, newly reported here. The QSRR model provided retention time predictions supporting the tentative annotation of eight rare acidic GSLs with insufficient MS/MS data. Among the identified species, Monosialogangliosides (GM) were the most abundant subclass, with GM1 and GM3 predominant. GD1a was the major disialoganglioside (GD) species, and the trisialoganglioside (GT), tetrasialoganglioside (GQ), and pentasialoganglioside (GP) subclasses were identified as GT1b, GQ1b, and GP1c, respectively. The STs were mainly of the SulfoHex type. O-acetylation was frequent in GQ species, while fucosylation and GalNAc attachment were primarily associated with GM and GD species. The sphingoid bases were mainly d18:0, d18:1, d20:0, and d20:1, while the N-acyl chains spanned C16:0–C24:0. For quantification, a method covering 17 subclasses enabled the simultaneous measurement of 89 GAs and 27 STs, representing the broadest quantification coverage in PD mouse brain studies to date. Further analysis revealed significant alterations in 20 GAs and 7 STs in PD, with GQ1b reported for the first time as a potential PD-related biomarker, and pathway analysis indicated disruption of b-series GA biosynthesis.

**Conclusions:**

This study provides a comprehensive profile of acidic GSLs in mouse brain tissues and reveals GQ alterations with pathway disruptions in PD, offering molecular evidence linking acidic GSL metabolism to this disease.

**Supplementary Information:**

The online version contains supplementary material available at 10.1186/s12944-026-02918-1.

## Introduction

Gangliosides (GAs) and sulfatides (STs), classes of acidic glycosphingolipids (GSLs), are characterized by the presence of at least one sialic acid (SA) residue or sulfate group [1]. Acidic GSLs are widely distributed but are especially abundant in the central nervous system (CNS) [2, 3], where they contribute to receptor signalling, cell–cell recognition, and membrane stability [4, 5]. Structural heterogeneity in glycan chains and ceramide moieties generates thousands of distinct species, and an aberrant GSL composition has been associated with several neurodegenerative diseases, including Parkinson’s disease (PD), Alzheimer’s disease (AD), and Huntington’s disease [6–8].

Given the extreme compositional complexity of tissue acidic GSLs and their overlapping physicochemical properties, robust structural characterization and accurate quantification remain challenging. Ultrahigh-performance chromatography coupled with mass spectrometry (MS) has been extensively employed for the analysis of acidic GSLs owing to its high resolution, sensitivity, accuracy, reproducibility, and ability to generate structure-specific fragments of both glycan chains and ceramide moieties [9–12]. Although high-resolution mass analysers, such as quadrupole-time-of-flight (Q-TOF) [13–15], Fourier transform ion cyclotron resonance [16], and Orbitrap [17], have been widely used, confident identification of complex acidic GSLs, particularly low-abundance species, remains challenging due to coeluting interferences. Recently, efficient separation techniques, such as liquid chromatography (LC) or ion mobility (IM) coupled with MS, have elicited breakthroughs in the identification of acidic GSLs, leading to the characterization of approximately 300 GAs thus far [18–20].

Chromatographic retention time (RT) is associated with lipid structural features and has been used to assist lipid identification [21–24]. Although various regression models have been developed to characterize acidic GSL retention behaviour under reversed-phase [15, 20, 25–27] and hydrophilic interaction LC [14] modes, they typically consider only single variables such as ceramide chain length. Given the pronounced structural heterogeneity of acidic GSLs, more advanced predictive models are warranted. Quantitative structure–retention relationship (QSRR) offers such potential and has been applied in metabolomics and lipidomics to aid compound identification [28–30]. However, its application to acidic GSLs remains limited, with only one study on monosialoganglioside 1 (GM1) species reported to date [15].

Beyond structural characterization, the quantitative analysis of acidic GSLs faces similar challenges. Triple quadrupole (QQQ) MS in multiple reaction monitoring (MRM) mode is widely used for GSL quantification because of its high specificity and throughput [31]. However, previous studies have focused mainly on abundant GM and disialoganglioside (GD) subclasses, whereas low-abundance but biologically important species, such as tetrasialoganglioside (GQ) types, remain difficult to quantify. Additionally, while GSLs are known to play an important role in PD, the exact mechanisms by which they contribute to PD pathogenesis remain unclear. Building on previous work, which improved the quantification of rare acidic GSLs [32], the present study applied an optimized method to quantify GSL alterations in PD mouse brains.

**Fig. 1:**
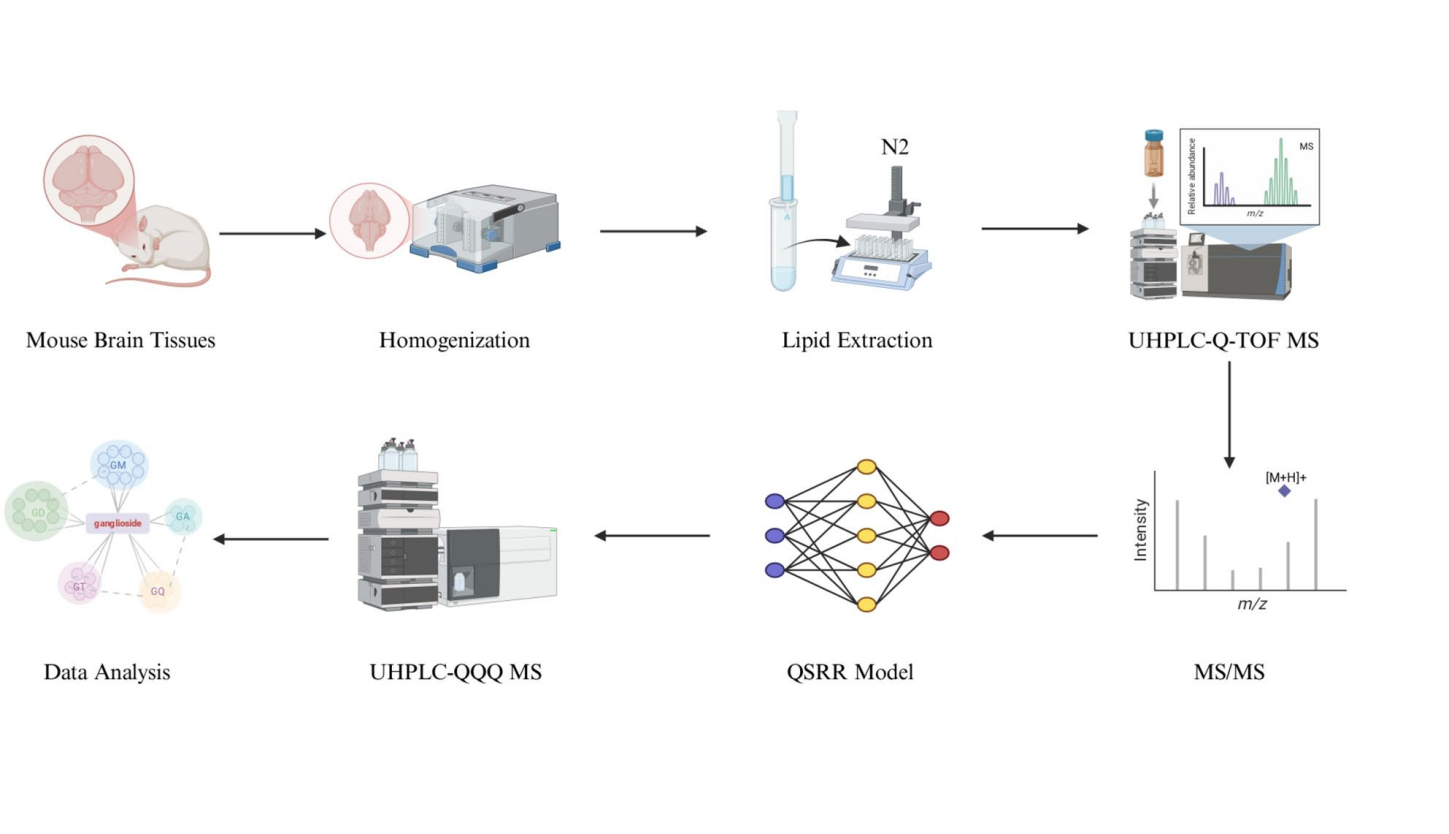
Workflow for acidic GSL profiling in mouse brain tissues

To address the current limitations in acidic GSL profiling, this study presents an integrated analytical strategy that combines prefractionation, UHPLC-Q-TOF MS characterization, QSRR modelling for retention time prediction, and enhanced dynamic multiple reaction monitoring (dMRM) quantification. This comprehensive approach significantly expands both the structural annotation and quantitative coverage of low-abundance GSLs, which have previously been difficult to analyze, thus providing a more complete understanding of the acidic GSL profile in mouse brain tissue. Compared with previous reports [26, 27, 33–36], this study offers a more extensive characterization of acidic GSL species. This analytical strategy was subsequently applied to PD mouse brain tissues to identify characteristic GSL alterations, providing potential biomarkers and mechanistic insights into PD pathogenesis (Fig. 1).

## Materials and methods

### Materials and chemicals

Sigma–Aldrich (St. Louis, MO, USA) provided GM1, GM2, GM3, GD1a, GD1b, and GD2 (all d18:1/18:0 species). Matreya LLC (State College, PA, USA) supplied GT1b and GQ1b (both d18:1/18:0 species), as well as sulfatide (d18:1/12:0). Cayman (Ann Arbor, MI, USA) provided the deuterated internal standard GM3-d_9_ (d18:1/16:0). J.T. Baker (Center Valley, PA, USA) supplied MS-grade methanol (MeOH), and RCI Labscan Limited (Bangkok, Thailand) provided HPLC-grade chloroform (CHCl_3_). Sigma–Aldrich supplied HPLC-grade formic acid (HCOOH) and MS-grade ammonium formate (NH_4_HCOO). A Milli-Q system (Millipore, MA, USA) generated ultrapure water (18.2 MΩ). Waters (Milford, MA, USA) provided Sep-Pak C_18_ cartridges (3 cc, 500 mg, 37–55 μm).

### Animals

C57BL/6 mice (*n* = 5) and A30P-synuclein transgenic mice (*n* = 7) were obtained from the Experimental Animal Center of Nanjing Medical University (Nanjing, China) and used as the healthy control (HC) and PD groups, respectively. The pathological validity of the A30P-synuclein transgenic mouse model was confirmed in previous research [37]. In addition, key pathological and behavioral features of PD were confirmed in the current cohort, including dopaminergic neuron loss, α-synuclein (α-Syn) aggregation, and motor coordination deficits, as shown in Fig. S1–S3. Each biological sample was prepared in triplicate for lipidomic analysis. All animal care and experimental procedures complied with the National Institutes of Health Guide for the Care and Use of Laboratory Animals and were approved by the Animal Protection and Ethics Committee of Nanjing Medical University. The mice were housed in a controlled environment (temperature: 23 ± 2 °C; humidity: 55 ± 5%; 12 h light/dark cycle) with free access to chow and water. After deep anaesthesia with isoflurane, the mice were transcardially perfused with precooled 1× PBS until the liver turned pale. The brains were removed, the skulls were stripped, and the intact tissues were stored at -80 °C in an ultralow-temperature freezer.

### Extraction and prefractionation of acidic GSLs

Acidic GSLs were extracted from mouse brain tissues and then prefractionated into 5 fractions by an improved strategy established in previous study [20]. In brief, 100 µL of mouse brain homogenate was mixed with 4 mL of CHCl_3_/MeOH (2:1, v/v) for lipid extraction, followed by orbital shaking for 20 min at ambient temperature. Subsequently, 0.8 mL of water was introduced into the sample mixture and vortexed for 5 min. Following centrifugation at 3,000 rpm for 15 min at 4 °C, the upper phase was removed, and the lower CHCl_3_ phase was further extracted with 0.9 mL of MeOH and 0.8 mL of water. Finally, both upper phases were pooled and evaporated to dryness under a gentle stream of nitrogen. The resulting residue was re-dissolved in 100 µL of MeOH and applied to a preconditioned C_18_ cartridge. Next, the C_18_ cartridge was stepwise eluted with MeOH at 20%, 40%, 50%, 70%, 75%, 80%, 85%, 90%, and 100% (3 mL per step) to fractionate acidic GSLs. Fractions corresponding to each GSL subclass were subsequently pooled to obtain extracts enriched in GM, GD, GQ/trisialoganglioside (GT), pentasialoganglioside (GP), and ST.

### UHPLC-Q-TOF MS conditions

Acidic GSLs were analyzed using a modified UHPLC–MS method as previously reported [20]. Chromatographic separation was carried out on an Agilent 1290 Infinity UHPLC system (Santa Clara, CA, USA) using an Agilent Zorbax Eclipse Plus C_18_ column (100 × 2.1 mm, 1.8 μm) maintained at 45 °C. The mobile phase consisted of (A) H_2_O and (B) MeOH, both of which contained 0.2% HCOOH and 10 mM NH_4_HCOO. A linear gradient at 0.4 mL/min was applied as follows: 80–85% B (0–3 min), 85–100% B (3–17 min), 100% B (17–19 min), decreased to 80% B (19–19.01 min), and maintained at 80% B (19.01–25 min). MS and MS/MS spectra were acquired on an Agilent 6550 ultrahigh-definition Q-TOF mass spectrometer in positive ion mode. The *m/z* scan ranges were set at 500–3000 for MS and 50–3000 for MS/MS. Collision energy (CE) was set at 15 and 45 V for targeted MS/MS acquisition.

### UHPLC-QQQ MS conditions and method validation

Quantitation was performed using an Agilent 6495 AJS-ESI QQQ MS operating in positive dMRM mode. The MRM analysis parameters were optimized as follows. The drying gas temperature was set at 150 °C with a flow rate of 14 L/min. The sheath gas temperature was set at 300 °C with a flow rate of 11 L/min. The nebulizer pressure was 25 psi, the capillary voltage was 5000 V, and the nozzle voltage was 1000 V. The RF voltage amplitude was set to 150 V for the high-pressure ion funnel and 40 V for the low-pressure ion funnel. The injection volume was 10 µL.

The peak areas were obtained using Agilent MassHunter Quantitative Analysis B.10.00 software. To mitigate the variability introduced by sample preparation and injection, relative quantification was performed by normalizing each compound’s peak area to the sum of all the detected GSLs within the same sample. This normalization strategy was adopted in light of the limited availability of stable isotope-labelled standards for most GSLs, and similar approaches have been reported in previous studies [38].

Method validation was performed using a quality control (QC) sample, and parameters such as linearity, limit of detection (LOD), limit of quantitation (LOQ), reproducibility, and recovery were assessed.

The GM3-d_9_ (d18:1/16:0) internal standard (IS) stock solution (212 µM) was diluted with MeOH to 9 different concentrations (0.0195, 0.0391, 0.0781, 0.156, 0.312, 0.625, 1.25, 2.5, and 5 µM) and injected for analysis. The data were imported using quantitative software, linear regression was performed by calculating the peak area response of the IS in the samples, and a calibration curve was constructed to evaluate the linearity. The linear range was defined as the concentration range with a regression coefficient *R*^2^ > 0.99.

The LOD and LOQ were determined by further diluting the lowest concentration in the calibration curve, while linearity was assessed using the correlation coefficient (*R*^2^). Specifically, the LOD and LOQ were defined as the lowest concentration that could be detected with a signal-to-noise ratio (S/N) of approximately 3 and 10, respectively.

Using the QC samples, precision for each GA or ST was assessed both intraday and interday to evaluate reproducibility. Intraday precision was evaluated on the basis of the relative standard deviation (RSD) of six replicates performed within one day, whereas interday precision was calculated using the RSD of nine replicates over three consecutive days.

Recovery was evaluated by calculating the ratio of the peak area of the IS spiked into the samples prior to extraction to that spiked after the extraction process. The IS stock solution (212 µM) was diluted to three final concentrations in the sample (0.0391, 0.312, and 2.5 µM for low, medium, and high levels, respectively). For each concentration level, triplicate samples were prepared.

MassHunter was used to import the data, extract the IS spiked before and after extraction, and compare their peak area responses.

### Data processing and statistics

#### MS data analysis

Data were processed using Agilent MassHunter Qualitative Analysis software (version B.10.00). Acidic GSLs in mouse brain samples were screened using the Find by Formula (FBF) algorithm. Matching parameters were specified as follows: an in-house curated acidic GSL database served as the reference formula library; up to five candidate formulas were permitted per detected feature; a mass tolerance of ± 15 ppm was applied; protonated (+ H) and sodiated (+ Na) adducts were considered in positive ion mode; charge states of 1–3 were permitted; and only identifications with a score greater than 70 were accepted. In addition, in silico MS/MS fragmentation patterns of glycosphingolipids were predicted using the LIPID MAPS glycosphingolipid MS/MS prediction tool (https://www.lipidmaps.org/tools/ms/SP_insilico_compare_form.php) to assist in structural annotation.

#### Dataset for QSRR analysis

Twenty-one high-abundance GAs identified in mouse brain tissues together with 8 GA standards were employed in GA model construction. The dataset was randomly split into a training set (23 GAs) and a prediction set (6 GAs) for developing the models and evaluating their predictability, respectively. Afterwards, a validation set (94 GAs), including the other 65 identified GAs from mouse brain tissues and the 29 GAs used in model construction, was employed as an external set to assess the predictive ability of the models. Similarly, 15 STs comprising 14 high-abundance STs identified in mouse brain tissues and 1 ST standard were employed in model construction for STs, and the dataset was randomly split into a training set (12 STs) and a prediction set (3 STs). Afterwards, a validation set (36 STs), including the other 21 identified STs from mouse brain tissues and the 15 STs used in model construction, was used as an external set for the evaluation of the predictive ability of the models.

#### Molecular descriptors and QSRR model statistics

**Fig. 2:**
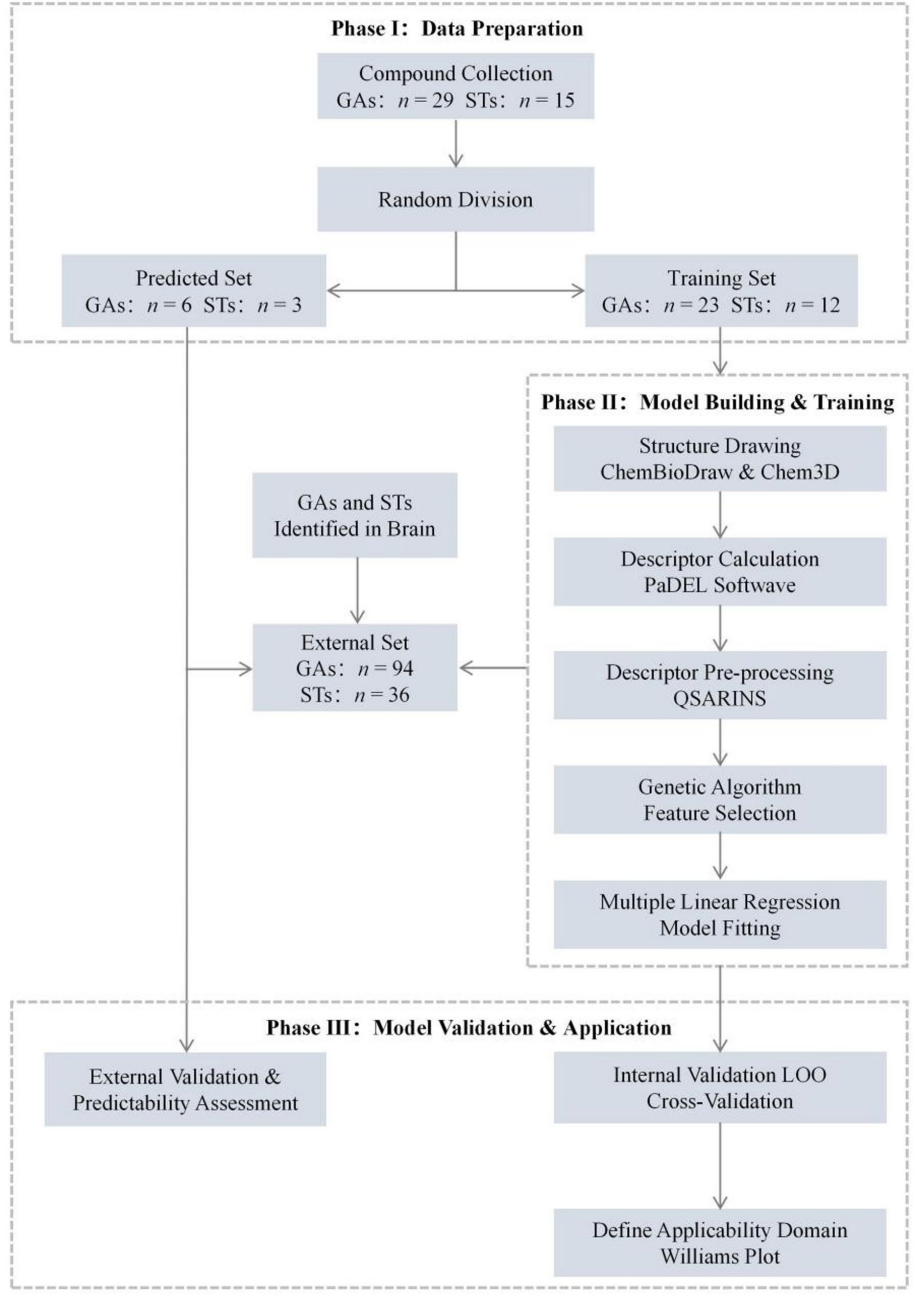
Workflow for the construction and validation of the QSRR models

The structures of acidic GSLs were drawn by ChemBioDraw and Chem3D software (Version 12.0), and molecular descriptors were calculated by PaDEL software. A preprocessing step was performed in QSARINS to remove constant, near-constant, and collinear descriptors (*r* > 0.9). Subsequently, a genetic algorithm–multiple linear regression (GA-MLR) model was constructed using QSARINS software, in which both the descriptor combinations and regression coefficients were optimized by tuning the genetic algorithm parameters (maximum number of descriptors = 4, number of generations = 10,000, population size = 1,000, mutation rate = 0.5). In addition, regression and validation metrics, including the squared correlation coefficient *R*^2^, adjusted *R*^2^, and root mean square error (RMSE), were employed to assess model performance [39]. These metrics reflect the model’s capacity to explain the variability observed in the experimental data. Typically, a higher correlation coefficient and a lower RMSE indicate better model performance. Correlation coefficient values approaching 1 indicate better model fit and suggest stronger predictive ability. Finally, the leave-one-out (LOO) cross-validation procedure was performed to validate the robustness of the GA-MLR models, and the applicability domain of the QSRR models was evaluated by leverage analysis expressed by a Williams plot. The overall workflow for the construction and evaluation of the QSRR models is illustrated in Fig. 2.

#### dMRM data analysis

The dMRM data were processed using Agilent MassHunter Quantitative Analysis software (version B.10.00) and subsequently analyzed in MetaboAnalyst (https://www.metaboanalyst.ca/). After centering and standardization, orthogonal partial least squares discriminant analysis (OPLS-DA) was performed to screen for differentially abundant GSLs between the two groups. GSLs with variable importance in projection (VIP) scores > 1 were selected as potential discriminative features. The closer the values of *R*^2^Y and Q^2^Y were to 1, the better the model fit. Heatmaps were constructed, and statistical analyses were conducted using GraphPad Prism 9.0. Group differences were assessed using independent-samples t tests or nonparametric Mann–Whitney U tests. Statistical significance was defined as *P* < 0.05. *P* values were adjusted for multiple testing using false discovery rate correction.

## Results and discussion

### UHPLC-Q-TOF MS profiling of acidic GSLs in mouse brain tissues

#### Identification of GAs

Acidic GSLs exhibit considerable structural heterogeneity and diversity in their glycan chains and ceramide moieties [2], resulting in the occurrence of numerous isobaric or isomeric species. Moreover, ion suppression from the matrix, nontargeted lipids or coeluted species, and interference signals from contaminants pose challenges to the detection of low-abundance GSLs [40]. In this study, the structural elucidation of acidic GSLs, particularly low-abundance isobaric or isomeric species, was significantly improved by adopting a prefractionation procedure and UHPLC-Q-TOF MS-based acidic GSL approach conducted in previous study [20].

**Fig. 3:**
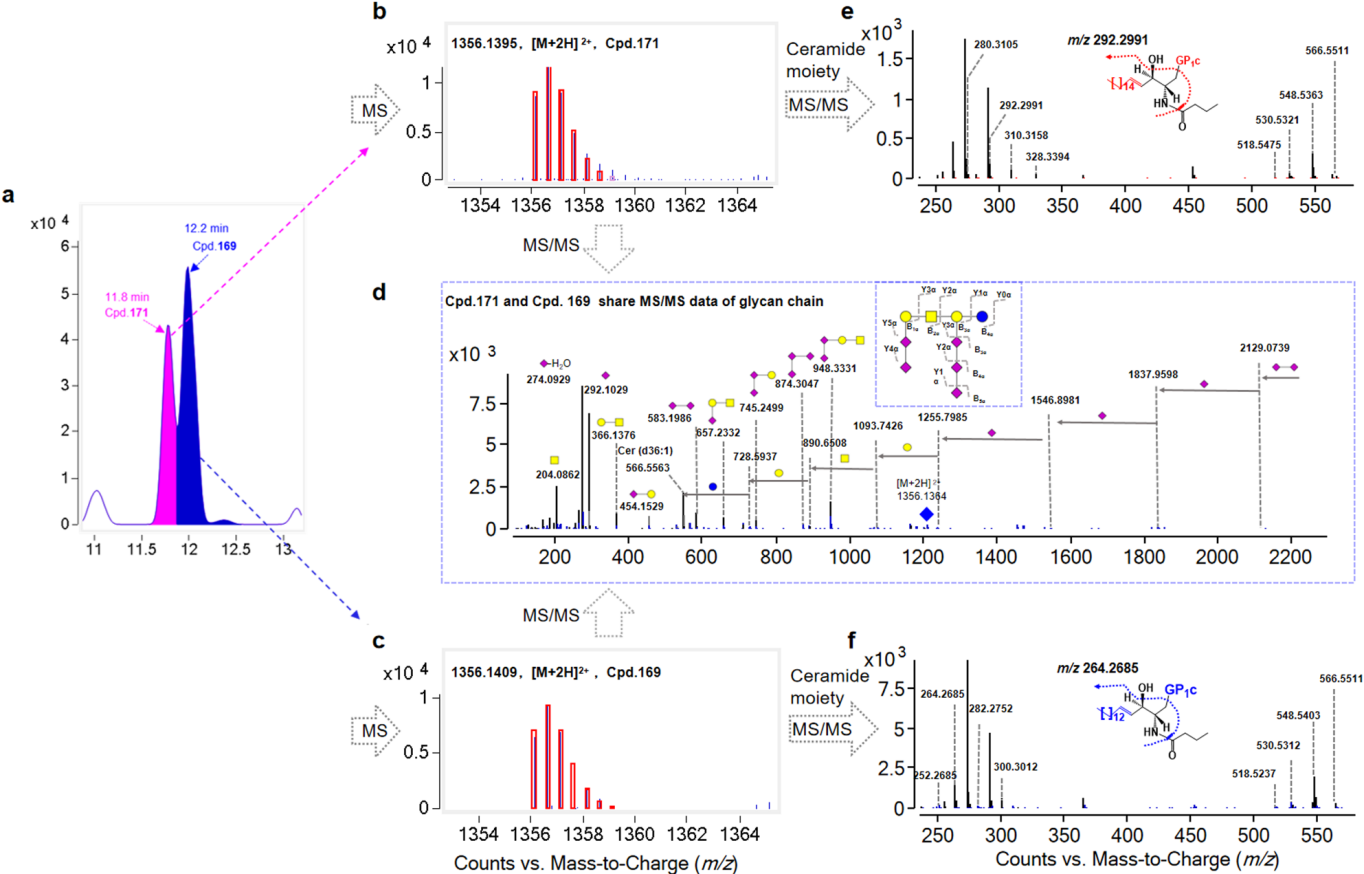
Differentiation of a pair of GA isomers, Cpd.169 and Cpd.171 (**a**); MS spectra of Cpd.171 (**b**) and Cpd.169 (**c**); MS/MS spectrum of the glycan chain of the two isomers (**d**); MS/MS spectra of the ceramide moiety of Cpd.171 (**e**) and Cpd.169 (**f**)

The structural elucidation of a pair of isomeric species, Cpd.169 and Cpd.171, is exemplified as follows. After matching with the established acidic GSL database, two peaks corresponding to the same molecular formula, C_117_H_199_N_7_O_63_, were obtained at 10.95 min and 11.59 min (Fig. 3a), owing to the [M + 2 H]^2+^ ion at *m/z* 1356.1395 and *m/z* 1356.1409, respectively (Fig. 3b–c). MS/MS analysis demonstrated that the compounds possessed similar glycan chains. As illustrated in Fig. 3d, five consecutive neutral losses of 291 Da were observed from *m/z* 2711.2656, yielding fragment ions at *m/z* 2129.0739, 1837.9598, 1546.8981, and 1255.7985, corresponding to the successive loss of five SA residues. These observations indicated that both compounds possessed five SA residues and hence belong to the GP type. Then, one neutral loss of 162 Da from *m/z* 1255.7985 to *m/z* 1093.7426, one neutral loss of 203 Da from *m/z* 1093.7426 to *m/z* 890.6508, and two neutral losses of 162 Da from *m/z* 890.6508 to *m/z* 728.5937 and *m/z* 566.5563 were detected, suggesting the presence of a saccharide chain composed of Hex–HexNAc–Hex–Hex on both compounds, hence belonging to the GP1 type. Moreover, the ions at *m/z* 583.1986 ([2*SA + H]^+^), *m/z* 745.2499 ([2*SA + Hex+H]^+^), *m/z* 874.3047 ([3*SA + H]^+^), and *m/z* 948.3331 ([2*SA + Hex+HexNAc + H]^+^) suggested that three consecutive SAs were linked on a Hex unit. Therefore, both compounds were identified as the GP1c type. Subsequently, their structures were further elucidated based on fragments arising from the ceramide moiety. As shown in Fig. 3e–f, ions at *m/z* 548.5363 ([M-glycan chain-H_2_O + H]^+^), *m/z* 530.5321 ([M-glycan chain-2*H_2_O + H]^+^), and *m/z* 518.5475 ([M-glycan chain-H_2_O-HCOH + H]^+^) were indicative of dihydroxylated Cer (d36:1) in both compounds. The ions at *m/z* 310.3158 ([d20:1-H_2_O + H]^+^), *m/z* 292.2991 ([d20:1–2*H_2_O + H]^+^), and *m/z* 280.3105 ([d20:1-H_2_O-HCOH + H]^+^) suggested a d20:1 sphingoid base on Cpd.171 (Fig. 3e). Moreover, the ions at *m/z* 282.2752 ([d18:1-H_2_O + H]^+^), *m/z* 264.2685 ([d18:1–2*H_2_O + H]^+^), and *m/z* 252.2685 ([d18:1-H_2_O-HCOH + H]^+^) indicated a d18:1 sphingoid base on Cpd.169 (Fig. 3f). Therefore, Cpd.169 was assigned as GP1c (d18:1/18:0), whereas Cpd.171 was assigned as GP1c (d20:1/16:0).

By using a similar strategy, 173 GAs were identified in mouse brain tissues. Notably, 13 isomeric GA pairs differing exclusively in glycan chains and another 13 differing solely in ceramide composition were reliably distinguished, as detailed in Table S1.

#### Identification of STs

**Fig. 4:**
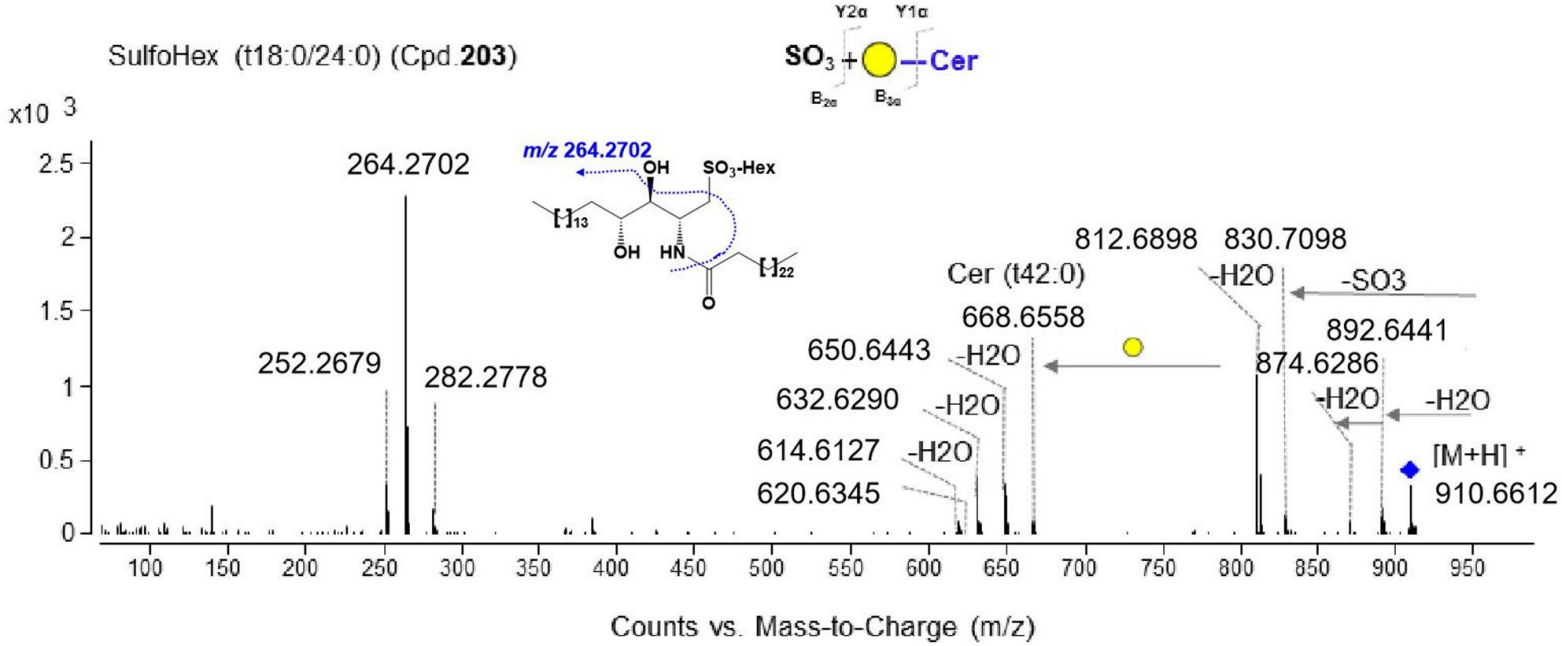
MS/MS spectrum of SulfoHex (t18:0/24:0) (Cpd.203)

The structural interpretation of STs was elucidated using an approach similar to that used for GAs. For example, on the basis of the [M + H]^+^ ion at *m/z* 910.6612, Cpd.203 was deduced as an ST with the formula C_48_H_95_NO_12_S. The glycan and ceramide structural features were then elucidated based on MS/MS data. First, an 80 Da neutral loss between *m/z* 910.6612 and *m/z* 830.7098 demonstrated the presence of a sulfate group. Second, a neutral loss of 162 Da from *m/z* 830.7098 to *m/z* 668.6558 suggested the loss of one hexosyl group. The ion at *m/z* 668.6558 ([M-glycan chain + H]^+^), along with the ions at *m/z* 650.6443 ([M-glycan chain-H_2_O + H]^+^), *m/z* 632.6290 ([M-glycan chain-2*H_2_O + H]^+^), *m/z* 620.6345 ([M-glycan chain-H_2_O-HCOH + H]^+^), and *m/z* 614.6127 ([M-glycan chain-3*H_2_O + H]^+^), indicated that Cpd.203 contained a trihydroxylated Cer (t42:0) as its ceramide moiety. Additionally, the ion at *m/z* 282.2778 corresponded to the loss of two H_2_O molecules from the sphingoid base, the ion at *m/z* 264.2702 was formed by the further loss of one H_2_O, and the ion at *m/z* 252.2679 was generated via the sequential loss of two H_2_O molecules and one formaldehyde (HCOH) from the sphingoid base. These fragments indicated a t18:0 sphingoid backbone for Cpd.203. Therefore, Cpd.203 was characterized as SulfoHex (t18:0/24:0) (Fig. 4).

Forty-seven STs were identified in mouse brain tissues based on the strategy described above. Notably, 8 ST isomer pairs differing in ceramide composition were reliably distinguished, as summarized in Table S1.

### Structural variation of acidic GSLs in mouse brain tissues

**Fig. 5:**
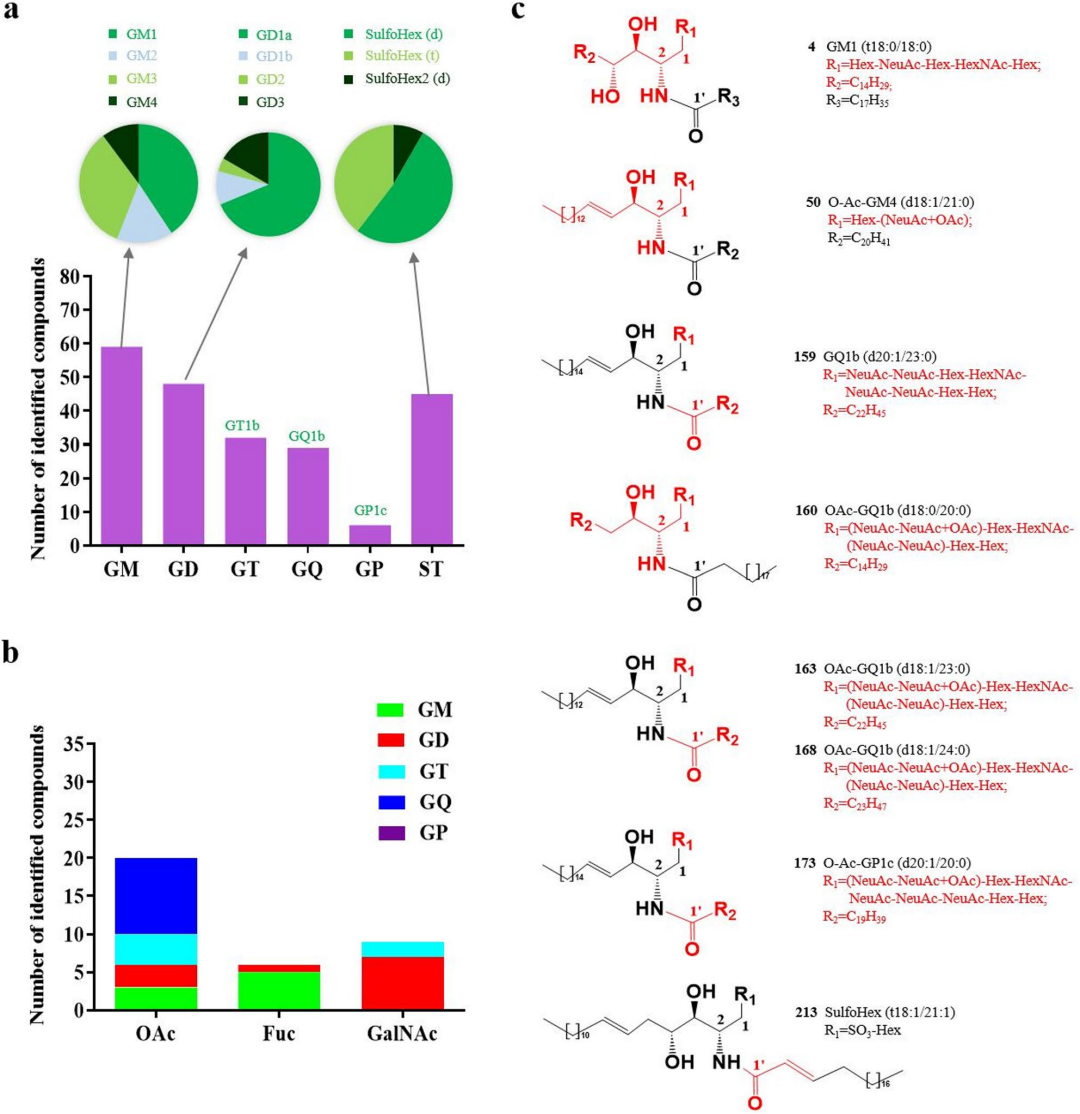
The number of different types of acidic GSLs identified in mouse brain tissues (**a**); the number of acidic GSLs with modifications (OAc, Fuc, and GalNAc) (**b**); and the structures of the 7 novel GAs and 1 novel ST (**c**)

In total, 220 acidic GSLs, including 173 GAs from 21 subclasses and 47 STs from 3 subclasses, were characterized in mouse brain tissues using MS and MS/MS analysis. To date, this study reports the greatest number of identified acidic GSLs from mouse brain tissues, with 75 GAs and 29 STs newly characterized. These structures have not been previously described in mouse brain tissues and are not recorded in existing lipid databases such as LIPID MAPS. As illustrated in Fig. 5a, GM was the dominant GA class in mouse brain tissues, with GM1 and GM3 accounting for the major species. Moreover, GD1a was the dominant GD subclass, while the GT, GQ, and GP subclasses were of the GT1b, GQ1b, and GP1c types, respectively. Moreover, the STs were mainly of the SulfoHex (d) type. Furthermore, Fig. 5b shows that O-acetylation predominated in the GQ type, whereas fucosylation and attachment of GalNAc were common in the GM and GD types, respectively.

The structural diversity of the identified GAs and STs, including GAs (GM, GD, GT, GQ, and GP; Table S2), O-acetylated GAs (Table S3), fucosylated GAs (Table S4), N-acetylgalactosamine GAs (Table S5), and STs (SulfoHex and SulfoHex2; Table S6), is summarized in Tables S2–S6. In these tables, the fatty acid chain length and degree of unsaturation are arranged by rows and the sphingoid backbones by columns, and the numbers indicate distinct glycan compositions (“1” = four sugars, “2” = three sugars, “3” = two sugars, and “4” = one sugar). The results indicated that d18:0, d18:1, d18:2, d20:0, and d20:1 were the dominant sphingoid backbones in mouse brain tissues, among which d18:1 was the most prevalent. The fatty acid chain ranged from 14 to 27 carbon atoms, and the degree of unsaturation ranged from 0 to 2. Among them, d16:0, d18:0, d20:0, d22:0, and d24:0 were the predominant types. Moreover, 8 previously unreported acidic GSLs, including 7 GAs (Cpd.4, 50, 159, 160, 163, 168, and 173) and 1 ST (Cpd.213), were characterized, and their structures are shown in Fig. 5c, with newly identified structures highlighted in red. The corresponding MS/MS spectra are provided in Fig. S4–S11.

The results revealed remarkable structural diversity of GSLs in mouse brain tissues, providing an essential foundation for further exploration of their biological functions. In the early stages of PD, the levels of protective complex GAs such as GM1, GD1a, GD1b, and GT1b progressively decrease in brain tissues, whereas simple GAs such as GM2 and GM3 abnormally accumulate [41–43]. Systemic deficiency of GM1 across both central and peripheral tissues in patients with PD has been reported, suggesting its potential as an early biomarker of neurodegenerative pathology [44, 45]. GM1 can bind to *α*-synuclein, maintaining its native helical conformation and preventing pathological aggregation; when the level of GM1 decreases, *α*-syn becomes more prone to misfolding and the formation of Lewy bodies [41]. Moreover, fluctuations in GM2 and GM3 levels may promote neuroinflammation and microglial activation, thereby accelerating neurodegenerative progression [42]. Moreover, in PD patients, the acyl-chain composition of ceramides in the anterior cingulate cortex shifts markedly towards shorter chains, suggesting that such remodelling of chain-length distribution may alter membrane fluidity and lipid-raft stability, consequently disturbing raft-dependent signal transduction and contributing to PD pathogenesis [46]. Therefore, comprehensive structural characterization of individual acidic GSLs in the central nervous system is highly important for elucidating their structure–function relationships in disease mechanisms, therapeutic development, and the discovery of diagnostic biomarkers.

### The established QSRR model supported the annotation of rare acidic GSLs

MS/MS fragment ions were pivotal in identifying acidic GSLs, especially for the discrimination of isomeric species. For instance, the fragment ion at *m/z* 657.2403 ([SA + Hex-HexNAc + H]^+^) is a diagnostic ion for GM1b, GD1a, GT1b, and GQ1c that differentiates them from GM1a, GD1b, GT1a, and GQ1a/GQ1b, respectively. Moreover, the fragment ion at *m/z* 948.3331 ([2*SA + Hex+HexNAc + H]^+^) is a diagnostic ion for GQ1b. Moreover, diagnostic fragment ions also aid in the identification of modified GAs, such as *m/z* 334.1125 ([OAc + SA+H]^+^) for O-acetylated GAs, *m/z* 512.1963 ([Fuc + Hex+HexNAc + H]^+^) for fucosylated GAs, and *m/z* 860.3146 ([HexNAc + Hex(SA)+HexNAc + H]^+^) for GAs with GalNAc attachment [20, 26, 36]. However, MS/MS fragments of some ultratrace-level acidic GSLs may fail to be detected [29]. When MS/MS fragment information is limited, retention time serves as orthogonal evidence complementary to MS data for compound identification. It helps narrow down candidate structures, particularly for low-abundance species with poor-quality MS/MS spectra. Several methods have been developed for RT prediction, and the QSRR models of GA and ST were constructed in this study.

#### QSRR modelling of GAs

The GA-MLR model was applied to build a QSRR model to predict the RTs of GAs. As a result, the best GA-MLR model was built on a general equation describing the negative logarithm of the compound RT (-log*t*_*R*_), with the regression equation containing two significant descriptors established as follows:

1 $$\begin{array}{ll}\mathrm{-log}{t}_{R}=-0.0193 \times \text {nAtomLC}\\-0.0072 \times \text {Xlog} P-0.3251\end{array}$$

where nAtomLC is the number of atoms in the largest chain and Xlog*P* is the octanol/water partition coefficient of the lipophilic compounds.

**Fig. 6:**
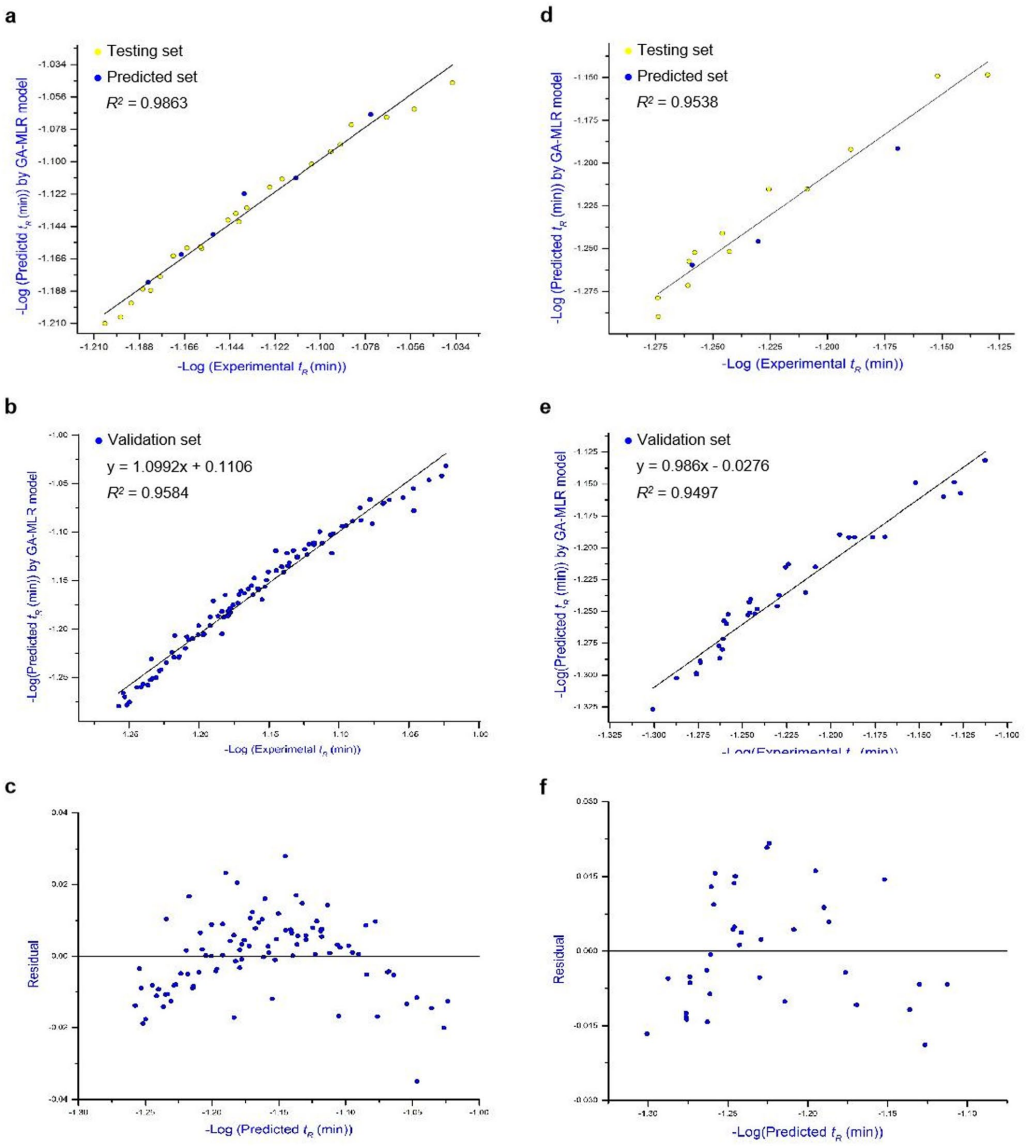
Plot of experimental versus QSRR-predicted RTs of GAs and STs in the modelling set (**a**, **d**) and the validation set (**b**, **e**), as well as the residual plot, which reveals the error range (**c** and **f**)

The plot of predicted RTs versus experimental RTs in the modelling set is illustrated in Fig. 6a, which indicates a good correlation (*R*^*2*^ = 0.9863). Ultimately, the predictive ability of the QSRR model was further assessed using a validation set composed of 94 GAs. The plot of predicted RTs versus experimental RTs in the validation set is illustrated in Fig. 6b, which also indicates a good correlation (*R*^*2*^ = 0.9584). Furthermore, the corresponding residual plot (Fig. 6c) was generated, indicating that the linearity of the equation was within the acceptable error range. The experimental and predicted RT results for each GA in the validation set are displayed in Table 1. Comparison between the predicted and the experimental results showed that most GAs exhibited accuracies between 97% and 103%, suggesting that the model is highly accurate in the prediction of GAs. Moreover, its ability to distinguish glycan chain isomers further supports the reliability of the developed QSRR model.

**Table 1:** Experimental versus QSRR-predicted retention time of the 94 gangliosides recruited in the validation set

Ganglioside	Retention time		Acc.(%)	Ganglioside	Retention time		Acc.(%)
	Exp.	Pred.			Exp.	Pred.	
GM1 (d18:0/16:0)	12.99	12.58	96.84	GD1a (d18:1/20:0)	14.39	14.39	100.00
GM1 (d18:0/18:0)	14.47	14.04	97.03	GD1a (d18:1/21:0)	15.11	15.19	100.53
GM1 (d18:0/22:0)	17.15	17.02	99.24	GD1a (d18:1/22:0)	15.72	16.03	101.97
GM1 (d18:1/14:0)	10.63	11.02	103.67	GD1a (d18:1/23:0)	16.37	16.92	103.36
GM1 (d18:1/16:0)	12.31	12.27	99.68	GD1a (d18:1/24:0)	16.87	17.45	103.44
GM1 (d18:1/17:0)	13.14	12.95	98.55	GD1a (d18:1/25:0)	17.44	18.21	104.42
GM1 (d18:1/18:0)	13.84	13.67	98.77	GD1a (d18:1/26:0)	17.85	18.99	106.39
GM1 (d18:1/19:0)	14.62	14.41	98.56	GD1b (d18:0/18:0)	13.97	13.16	94.20
GM1 (d18:1/20:0)	15.26	15.21	99.67	GD1b (d18:0/20:0)	15.48	14.83	95.80
GM1 (d18:1/21:0)	15.87	16.06	101.20	GD1b (d18:1/16:0)	11.71	11.77	100.51
GM1 (d18:1/22:0)	16.52	16.95	102.60	GD1b (d18:1/17:0)	12.52	12.42	99.20
GM1 (d18:1/23:0)	17.17	17.89	104.19	GD1b (d18:1/18:0)	13.32	13.11	98.42
GM1 (d18:1/24:0)	17.96	18.45	102.73	GD1b (d18:1/19:0)	14.14	13.84	97.88
GM1 (d18:1/16:1)	11.13	11.97	107.55	GD1b (d18:1/20:0)	14.84	14.60	98.38
GM1 (d18:1/17:1)	11.92	12.34	103.52	GD1b (d18:1/21:0)	15.56	15.41	99.04
GM1 (d18:1/18:1)	12.74	13.24	103.92	GD1b (d18:1/22:0)	16.12	16.26	100.87
GM1 (d18:1/20:1)	14.29	14.78	103.43	GD1b (d18:1/23:0)	16.72	17.16	102.63
GM1 (d18:1/21:1)	15.26	16.03	105.05	GD1b (d18:1/24:0)	17.24	18.11	105.05
GM1 (d18:1/24:1)	17.01	17.79	104.59	GD1b (d18:1/25:0)	17.77	18.85	106.08
GM2 (d18:1/16:0)	12.77	12.68	99.30	GD2 (d18:1/18:0)	13.67	13.54	99.05
GM2 (d18:1/17:0)	13.48	13.38	99.26	GD2 (d18:1/20:0)	15.06	15.08	100.13
GM2 (d18:1/18:0)	14.19	14.12	99.51	GD3 (d18:1/18:0)	13.79	13.85	100.44
GM2 (d18:1/19:0)	14.87	14.90	100.20	GD3 (d18:1/19:0)	14.50	14.61	100.76
GM2 (d18:1/20:0)	15.56	15.72	101.03	GD3 (d18:1/20:0)	15.22	15.42	101.31
GM2 (d18:1/21:0)	16.21	16.59	102.34	GalNAc-GD1a (d18:1/16:0)	11.14	11.35	101.89
GM2 (d18:1/22:0)	16.92	17.51	103.49	GalNAc-GD1a (d18:1/18:0)	12.71	12.63	99.37
GM2 (d18:1/23:0)	17.38	18.06	103.91	GalNAc-GD1a (d18:1/19:0)	13.48	13.34	98.96
GM2 (d18:1/24:0)	17.91	18.63	104.02	GalNAc-GD1a (d18:1/20:0)	14.22	14.34	100.84
GM3 (d18:0/18:0)	14.79	14.49	97.97	GalNAc-GD1a (d18:1/21:0)	14.99	14.97	99.87
GM3 (d18:0/20:0)	16.17	16.14	99.81	GT1b (d18:0/18:0)	13.12	12.97	98.86
GM3 (d18:1/16:0)	13.23	12.96	97.96	GT1b (d18:0/20:0)	14.54	14.30	98.35
GM3 (d18:1/17:0)	13.82	13.68	98.99	GT1b (d18:0/22:0)	15.86	15.73	99.18
GM3 (d18:1/18:0)	14.37	14.43	100.42	GT1b (d18:1/16:0)	10.86	11.12	102.39
GM3 (d18:1/19:0)	15.06	15.23	101.13	GT1b (d18:1/17:0)	11.69	11.74	100.43
GM3 (d18:1/20:0)	15.74	16.07	102.10	GT1b (d18:1/18:0)	12.44	12.39	99.60
GM3 (d18:1/21:0)	16.39	16.96	103.48	GT1b (d18:1/19:0)	13.27	13.28	100.08
GM3 (d18:1/22:0)	17.11	17.82	104.15	GT1b (d18:1/20:0)	13.96	13.80	98.85
GM3 (d18:1/23:0)	17.56	18.21	103.70	GT1b (d18:1/21:0)	14.71	14.56	98.98
GM3 (d18:1/24:0)	18.09	19.04	105.25	GT1b (d18:1/22:0)	15.36	15.37	100.07
GD1a (d18:0/16:0)	11.96	11.65	97.41	GT1b (d18:1/23:0)	16.02	16.22	101.25
GD1a (d18:0/18:0)	13.57	13.16	96.98	GT1b (d18:1/24:0)	16.57	16.75	101.09
GD1a (d18:0/20:0)	15.18	14.62	96.31	GQ1b (d18:1/16:0)	10.56	10.75	101.80
GD1a (d18:0/22:0)	16.49	16.10	97.63	GQ1b (d18:1/18:0)	12.16	11.89	97.78
GD1a (d18:1/16:0)	11.33	11.60	102.38	GQ1b (d18:1/20:0)	13.71	13.24	96.57
GD1a (d18:1/17:0)	12.14	12.25	100.91	GQ1b (d18:1/22:0)	15.11	15.36	101.65
GD1a (d18:1/18:0)	12.94	12.92	99.85	GP1c (d18:1/18:0)	11.59	11.67	100.69
GD1a (d18:1/19:0)	13.69	13.64	99.63	GP1c (d18:1/20:0)	13.12	12.91	98.40

*Abbreviations*: *Exp.* Experimental, *Pred.* Predicted, *Acc.* accuracy

Acc. (%) = Pred. / Exp. × 100%

#### QSRR modelling of STs

In a similar manner, the GA-MLR model was applied to build a QSRR model to predict the RTs of STs. As a result, the best GA-MLR model was obtained using three descriptors, as shown in the following equation:

2 $$\begin{array}{ll}\mathrm{-log}{t}_{R}=-34.7288 \times \text {AATSC6e}-0.0037\\\times \text {MDEC}\_23-0.0001 \times \text {VR}1_\text D -0.6627\end{array}$$

where AATSC6e is a Broto–Moreau autocorrelation descriptor, defined as the average centred Broto–Moreau autocorrelation of lag 6 weighted by Sanderson electronegativities, indicating a correlation between polarity and topological structure; MDEC_23 is defined as the molecular distance edge between all secondary and tertiary carbons, providing information on ceramide moiety properties; and VR1_D_, a topological distance matrix descriptor, is defined as a Randic-like eigenvector-based index derived from the topological distance matrix.

The plot of predicted RTs versus experimental RTs in the modelling set is illustrated in Fig. 6d, which indicates a good correlation (*R*^*2*^ = 0.9538). Finally, the predictive performance of the QSRR model was further assessed using a validation set composed of 36 STs. The plot of predicted RTs versus experimental RTs in the validation set is illustrated in Fig. 6e, which also indicates a good correlation (*R*^2^ = 0.9497); the corresponding residual plot demonstrated that the linearity of the equation was within the acceptable error range (Fig. 6f). The experimental and predicted RTs for each ST in the validation set are displayed in Table 2, in which most STs displayed accuracies between 97% and 105%, supporting the high predictive accuracy of the model for STs.

**Table 2:** Experimental versus QSRR-predicted retention time of the 36 sulfatides recruited in the validation set

Sulfatide	Retention time		Acc.(%)	Sulfatide	Retention time		Acc.(%)
	Exp.	Pred.			Exp.	Pred.	
SulfoHex (d18:0/16:0)	18.21	18.09	99.34	SulfoHex2 (d18:1/24:0)	18.21	18.09	99.34
SulfoHex (d18:0/18:0)	15.01	15.55	103.60	SulfoHex (d18:1/25:1)	18.25	18.70	102.47
SulfoHex (d18:0/22:0)	17.65	17.89	101.36	SulfoHex (d18:1/26:1)	18.89	19.56	103.55
SulfoHex (d18:0/23:0)	18.31	19.14	104.53	SulfoHex (t18:0/23:0)	17.59	17.40	98.92
SulfoHex (d18:0/24:0)	18.89	19.92	105.45	SulfoHex (t18:0/24:0)	18.23	18.69	102.52
SulfoHex (d18:1/16:0)	13.49	13.82	102.45	SulfoHex (t18:0/25:0)	18.87	19.51	103.39
SulfoHex (d18:1/17:0)	14.19	14.10	99.37	SulfoHex (t18:1/17:0)	13.38	14.36	107.32
SulfoHex (d18:1/18:0)	14.77	15.19	102.84	SulfoHex (t18:1/20:0)	15.37	15.55	101.17
SulfoHex (d18:1/19:0)	15.48	15.56	100.52	SulfoHex (t18:1/22:0)	16.75	16.33	97.49
SulfoHex (d18:1/20:0)	16.17	16.41	101.48	SulfoHex (t18:1/23:0)	17.44	17.70	101.49
SulfoHex (d18:1/21:0)	16.81	17.19	102.26	SulfoHex (t18:1/24:0)	18.11	17.88	98.73
SulfoHex (d18:1/22:0)	17.49	17.86	102.12	SulfoHex (t18:1/25:0)	18.78	19.49	103.78
SulfoHex (d18:1/23:0)	18.15	18.18	100.17	SulfoHex (t18:1/18:1)	12.96	13.53	104.40
SulfoHex (d18:1/24:0)	18.79	19.45	103.51	SulfoHex (t18:1/22:1)	15.67	15.48	98.79
SulfoHex (d18:1/25:0)	19.38	20.07	103.56	SulfoHex (t18:1/23:1)	16.38	17.18	104.88
SulfoHex (d18:1/26:0)	19.99	21.22	106.15	SulfoHex (t18:1/24:1)	16.99	17.62	103.71
SulfoHex (d18:1/23:1)	16.95	17.27	101.89	SulfoHex (t18:1/25:1)	17.61	17.83	101.25
SulfoHex (d18:1/24:1)	17.62	17.48	99.21	SulfoHex (t18:1/26:1)	18.33	18.70	102.02

#### Application of the established QSRR model for annotating acidic GSLs lacking sufficient MS/MS fragments

In this study, during the identification of acidic GSLs in mouse brain tissues, it was also found that the MS data for several rare acidic GSLs were inadequate for rigorous characterization. Therefore, QSRR-derived retention time prediction helps prioritize candidate structures from high-resolution MS-based formula matching, increasing confidence in the annotation. For instance, after matching with an in-house acidic GSL database, the peak at 16.67 min corresponded to the molecular formula C_48_H_91_NO_12_S. An 80 Da neutral loss from *m/z* 906.6335 to *m/z* 826.6790 was demonstrated to be an ST. No ions that indicated the ceramide moiety were detected, but ions at *m/z* 308.3059 (t20:1–2*H_2_O + H]^+^) and *m/z* 292.2991 (d20:1–2*H_2_O + H]^+^) implied that this peak might be SulfoHex (d20:1/23:0) or SulfoHex (t20:1/22:1). Afterwards, the structures of these two possible STs were drawn, and their molecular descriptors were calculated, which were subsequently imported into the established QSRR modelling of STs to predict their RTs. Finally, the predicted RT of SulfoHex (t20:1/22:1) was 16.67 min. Consequently, the peak at 16.67 min was identified as SulfoHex (t20:1/22:1) (Cpd. 220). While platforms such as GNPS enable compound annotation via spectral similarity, their utility is often limited by the sparse spectral coverage of rare or low-abundance species such as GAs and STs. In contrast, the QSRR model offers retention time as supporting evidence when fragment ion information is incomplete, enhancing the reliability of the annotation of structurally complex and low-abundance GSLs.

### Relative quantification of acidic GSLs in mouse brain tissues

#### MRM analysis of acidic GSLs

##### Optimization of MRM transitions for acidic GSLs

Selecting MRM transitions is critical for accurate quantification. By selecting the most abundant and specific product ions derived from the specific precursor ion, this approach enhances the S/N and reduces interference from coeluting compounds, thereby improving the specificity, accuracy, and reproducibility of the method.

The optimization procedure for the MRM method is described below. Five d18:1/18:0 species (GM3, GD1b, GT1b, GQ1b, and SulfoHex), identified in the QC samples using high-resolution MS and MS^2^ data, were employed as representative GAs and STs for optimization.

The selection of product ions was guided by both experimental optimization and literature precedent. In previous GA studies, *m/z* 292.1 ([SA + H]^+^) and *m/z* 274.1 ([SA-H₂O + H]^+^) were employed as characteristic fragment ions for sialylated GAs [32, 47]. In this study, *m/z* 274.1 exhibited higher response intensity and lower background noise across all subclasses and was therefore selected as the primary product ion for quantitative analysis. With respect to STs, SulfoHex predominantly generated the characteristic ion [M-SO_3_-H_2_O]^+^, which is consistent with the fragment losses observed in previous studies and was therefore selected for MRM quantification [32, 48]. Although different GAs yield specific fragment ions corresponding to individual structural features, the ions at *m/z* 316.1 (from O-acetylation), 512.2 (from fucosylation), 860.3 (from GalNAc), and 274.1 showed high abundance. Therefore, the ion at *m/z* 274.1 was chosen as the product ion for the MRM transitions of GM, GD, GT, and GQ. Ions at *m/z* 316.1, 512.2, and 860.3 were chosen for OAc-GAs, Fuc-GAs, and GalNAc-GAs, respectively. The CE was subsequently optimized for the previously selected transition pairs to achieve optimal peak shape and signal response.

##### Establishment of a dMRM method for quantification

**Fig. 7:**
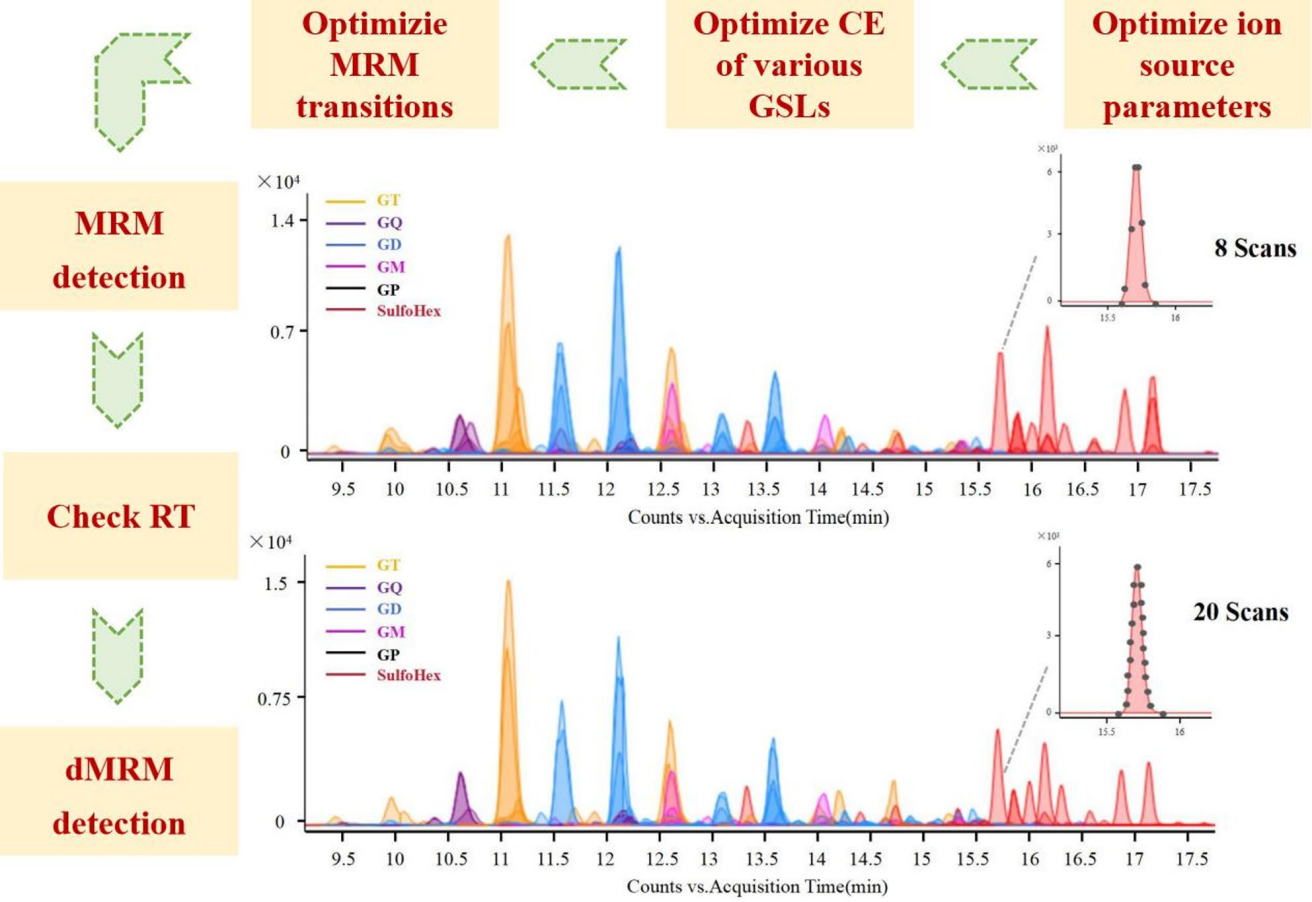
Development procedure for the MRM detection method of acidic GSLs in mouse brain tissues and the MRM chromatograms obtained by using routine MRM (upper) and dMRM methods (lower)

A total of 220 MRM transitions were initially selected for analysis. However, the large number of transitions significantly reduced the dwell time per ion, leading to poor peak shapes and low signal responses. Therefore, a dMRM method was adopted. RTs of the compounds were confirmed and used to schedule their respective acquisition windows (0.07 min) in the dMRM method, with a cycle time of 500 ms. The dMRM method markedly enhanced chromatographic peak definition and signal intensity, particularly for low-abundance species or analytes with low ionization efficiency, and therefore contributed to improved detection sensitivity and quantification precision (Fig. 7).

**Fig. 8:**
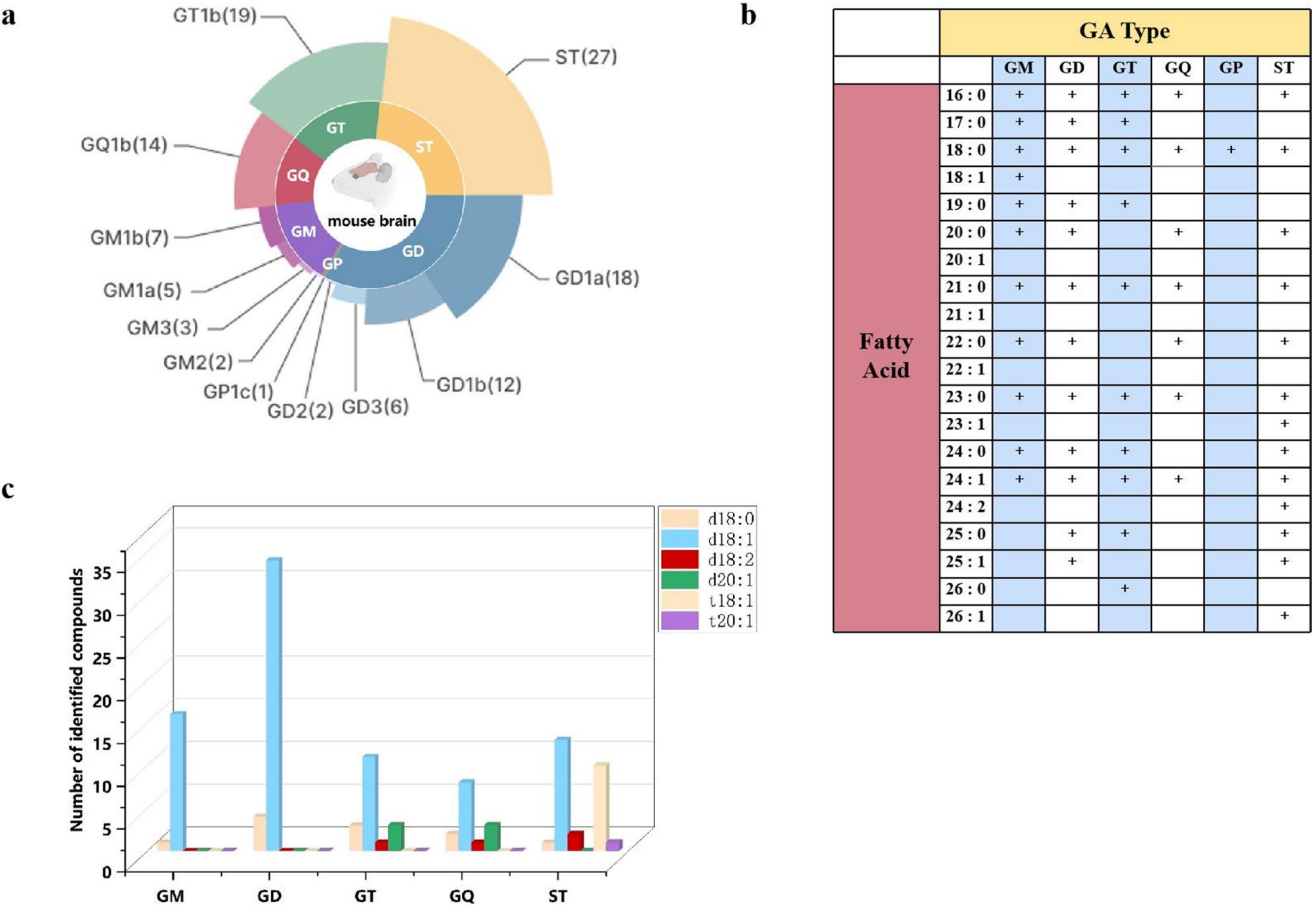
Structural diversity of acidic GSLs in the brain tissues of PD mice. The number of GA subclasses and STs (**a**); the distribution of GAs and STs bearing distinct fatty acid chains in mouse brain tissues (**b**); and the number of GAs and STs with different sphingoid bases (**c**)

By using the optimized dMRM method, together with the employment of IS, 116 acidic GSLs in mouse brain tissues were quantified. These included 38 compounds from the GD type, 17 from the GM type, 19 from the GT type, 14 from the GQ type, 1 from the GP type, and 27 from the SulfoHex type (Fig. 8a). To further characterize the structural diversity of these lipids, their fatty acid chain lengths and sphingoid backbones were analysed. Figure 8b shows that the fatty acid chain lengths ranged from 16 to 26, with 0–2 degrees of unsaturation. The most common types are 16:0, 18:0, 21:0, 23:0, and 24:1. Moreover, d18:0 and d18:1 serve as the primary sphingoid backbones of the GAs, whereas d18:1 and t18:1 are the main backbones of the STs (Fig. 8c).

Subsequent analysis of the GA subclass distribution in PD mouse brain tissues revealed that GD, GT, and GM were the most dominant types, collectively accounting for 83% of the total GA species. A general rank order of GD > GT > GM > GQ > GP was observed, with the most abundant GD species being 176 times more abundant than the least abundant GQ species.

#### Method validation

The method validation results demonstrated good performance across multiple parameters. The calibration curve for GM3-d_9_, as shown in Table 3, exhibited strong linearity, with a correlation coefficient (*R*²) ≥ 0.9923 over a range of more than two orders of magnitude, and the LOQ ranged from 0.0195 to 5 µM.

**Table 3:** Linear equation, correlation coefficient (*R*^2^), linear range, LOQ, and LOD of GM3-d_9_

IS	Calibration equation	*R* ^2^	Linear range (µM)	LOD (µM)	LOQ (µM)
GM3-d_9_ (d18:1/16:0)	Y = 2941.7X + 377.26	0.9923	0.0195 ~ 5	0.00065	0.00195

Reproducibility was assessed through the determination of RSD values for all the GSLs in the samples. As shown in Fig. S12, approximately 80% of the GSLs in the QC samples presented intraday precision RSD values less than 20%, and more than 60% presented interday RSD values less than 20%, indicating that the method demonstrates good precision for the quantitative analysis of GSLs. Recovery tests for the IS GM3-d_9_ (d18:1/16:0) at low, medium, and high concentrations, as shown in Table 4, yielded recovery rates of 98.33%, 102.59%, and 96.89%, respectively, with an average recovery of 99.27%. These results indicate that the method is efficient at recovering spiked samples.

**Table 4:** Recovery rate of GM3-d_9_

IS	Low# Recovery (%)	Medium& Recovery (%)	High* Recovery (%)	Mean Recovery (%)
GM3-d_9_ (d18:1/16:0)	98.33	102.59	96.89	99.27

Low^#^, low concentration (0.0391 µM)

Medium^&^, medium concentration (0.312 µM)

High^*^, high concentration (2.5 µM)

In summary, the validated method exhibits excellent linearity, reproducibility, and recovery, making it suitable for the quantification of GSLs in complex samples.

### Exploration of PD-related GAs and STs

PD is a common neurodegenerative disease that mainly impacts those above 60 years of age, with a steadily increasing incidence due to population ageing [49]. Although the exact aetiology and pathogenesis remain unclear, PD is widely believed to arise from a combination of genetic predisposition and external influences [49]. Pathologically, it is exhibited by the aggregation of *α*-syn in the form of Lewy bodies, reflecting its classification as a synucleinopathy [50]. Despite notable advances in elucidating the underlying mechanisms of PD, clinical diagnosis still largely depends on motor symptoms emerging in the mid-to-late stages, whereas the identification and validation of clinically applicable biomarkers remain major challenges [51].

**Fig. 9:**
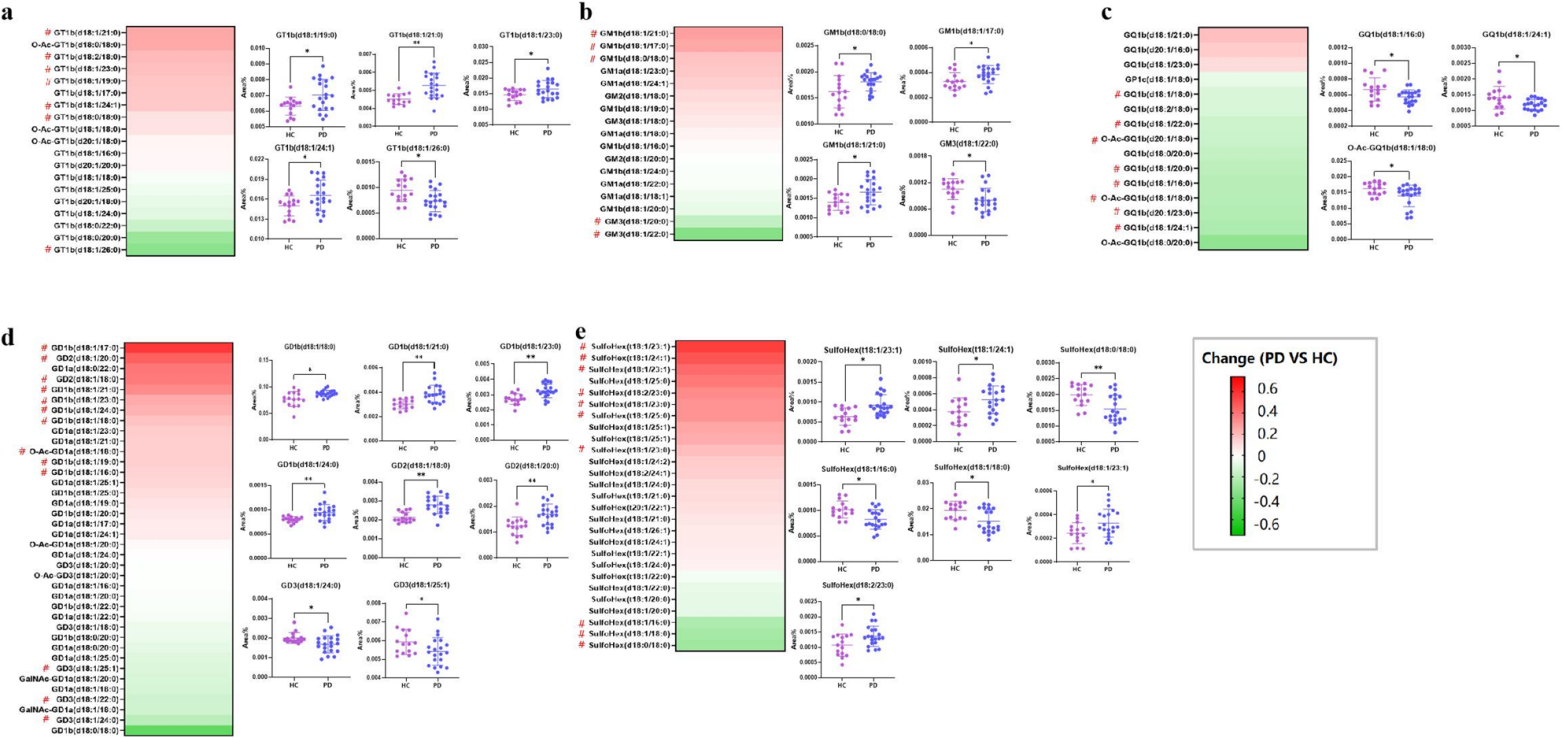
Heatmaps illustrating the changes and *P* values of acidic GSL signatures between the HC and PD groups. GT type (**a**); GM type (**b**); GQ type (**c**); GD type (**d**); and ST type (**e**). Change is calculated as (PD level - HC level)/HC level. Positive values indicate increased GSL levels in the PD group relative to those in the HC group, whereas negative values reflect decreased levels in the PD group relative to those in the HC group. Signatures with VIP > 1 identified by multivariate analysis are labelled with “#” as potential features for distinguishing the PD and HC groups. Twenty GAs and seven STs were differentially expressed in the PD group. (PD group, *n* = 7; HC group, *n* = 5; each sample was prepared in triplicate). Samples were compared using the independent-samples t test or the Mann–Whitney U test. *, *P* < 0.05; **, *P* < 0.01

Given the essential role of GSLs in maintaining neuronal membrane integrity, signal transduction, and synaptic function, increasing evidence suggests their involvement in the pathogenesis of neurodegenerative diseases including PD [42]. To further investigate the dysregulation of GSLs in PD, distribution patterns of acidic GSLs were analysed in brain tissues from PD model mice and HCs. Heatmap visualization revealed the difference in GSL levels between the HC and PD groups. The relative changes in the GSL levels were calculated with the following equation: Change = (PD level - HC level)/HC level. The analysis revealed that more than 50% of the GT, GM, GD, and ST types were elevated in the PD group versus the HC group. In contrast, more than 80% of the GQ and GP types were downregulated in the PD group (Fig. 9).

To explore potential molecular signatures related to PD mice, multivariate analysis was conducted on GSL profiles from PD and HC mice. First, OPLS-DA was performed for the classification (Fig. S13). The *R*^2^Y value of the OPLS-DA model validation chart was 0.951, and the Q^2^ value was 0.767, indicating that the GSLs in the PD group were markedly different from the HC group. Variables with VIP values greater than 1.0 were selected as candidate molecular markers, as depicted in Fig. 9. Overall, 43 GAs were screened, with 13 GD, 5 GM, 8 GQ, 7 GT, and 10 ST species. Additionally, an independent-samples t test or the Mann–Whitney U test was conducted to assess significant univariate differences among the candidate markers between groups. In this model, 27 compounds showed significant differences (*P* < 0.05), including 3 GM1b, 1 GM3, 4 GD1b, 2 GD2, 2 GD3, 5 GT1b, 2 GQ1b, 1 O-Ac-GQ1b, and 7 STs, as shown in Tables S7–S8.

These potential features showed that, in comparison to the control group, the highly abundant GM1b, GD1a, and GT1b in PD brain tissues were downregulated or remained relatively stable, whereas GQ1b showed an overall downward trend. These findings are in agreement with earlier studies reporting the downregulation of complex GAs such as GM1a, GD1a, GD1b, and GT1b in the brain tissues of PD model mice [52]. While the findings are robust and statistically significant within this cohort, further validation in a larger, independent animal cohort would help to enhance the generalizability of the results.

GM1 is believed to exert a wide range of neuroprotective effects. It can bind to *α*-syn to prevent its fibrosis and pathological accumulation. Subcutaneous administration of GM1a is considered a promising therapeutic approach for PD, as clinical trials have indicated its potential to slow disease progression.

GD1b has been shown to have antiapoptotic effects on hippocampal neurons [53], and it can activate bradykinin B2 receptors, thus promoting neuronal differentiation and maturation through calmodulin kinase II-dependent pathways [54]. In this study, high-abundance GD1a was downregulated, while several GD1b species, including GD1b (d18:1/18:0), GD1b (d18:1/21:0), GD1b (d18:1/23:0), and GD1b (d18:1/24:0), were elevated. In the brains of *St3gal2* knockout mice, a similar alteration pattern characterized by reduced levels of GD1a and GT1b, together with elevated levels of GM1 and GD1b, has been reported. These changes suggest the possible presence of compensatory mechanisms mediated by other sialyltransferases [55]. Furthermore, elevated plasma GD1b levels have been observed in PD patients [56], although interstudy variability may be attributed to differences in disease models, sample types, and analytical approaches.

Unlike the neuroprotective effects of GM1 and GD1b, GT1b has been shown to induce dopaminergic neuron death in the substantia nigra by activating microglia and promoting nitric oxide release [57]. Interestingly, nanomolar concentrations of GT1b oligosaccharide moieties can also promote dendritic growth in neuroblastoma cells, as well as in hippocampal and Purkinje neurons [58]. The dual roles of GT1b highlight the complexity of GA function in neurodegenerative settings.

In this model, polysialylated GAs, including GQ1b (d18:1/16:0), GQ1b (d18:1/24:1), and O-Ac-GQ1b (d18:1/18:0), were found to be significantly downregulated—these findings, to the best of the authors’ knowledge, have not been previously reported. Although GQ1b is a structurally complex, low-abundance b-series GA, the method enabled its stable and reliable detection, highlighting its potential as a sensitive disease indicator. It contains a long oligosaccharide chain and four sialic acid residues, which are thought to confer a high net negative charge and may affect its localization within membrane microdomains as well as interactions with membrane-associated proteins. GQ1b enhances neurite outgrowth and synaptic plasticity by modulating NMDA receptor activity and Ca²⁺ influx and has been reported to promote BDNF expression more effectively than GM1 [59, 60]. It also modulates 5-HT1 receptor affinity, contributing to improved cognitive functions such as learning and memory [61, 62]. Its downregulation may attenuate neurotrophic signalling pathways and impair calcium homeostasis, thereby contributing to neurodegeneration in PD. Exogenous GQ1b has been shown to restore BDNF expression, reduce *Aβ* and tau pathology, and improve cognitive decline in AD models [60]. Given the shared involvement of BDNF dysregulation across neurodegenerative disorders, GQ1b may likewise exert neuroprotective effects in PD. Additionally, decreased expression of O-acetyl-GQ1b was observed in PD mouse brain tissue. 9‑O‑acetylation of GAs has been shown to influence their metabolic stability, membrane microdomain localization, and cell‑surface interactions, thereby contributing to immune regulation and neurodevelopment [63]. In GD3, 9-O-acetylation has been reported to attenuate pro-apoptotic mitochondrial signalling, promoting cell survival [64]. By analogy, O-acetyl-GQ1b may contribute to neuronal survival and synaptic maintenance, although its precise role in PD pathogenesis remains unclear. Collectively, the observed downregulation of GQ1b and its O-acetylated derivatives suggests their potential not only as biomarkers for PD but also as molecules of therapeutic relevance, warranting further mechanistic investigation.

In addition to GAs, marked alterations in ST composition were also observed. Long-chain unsaturated species such as SulfoHex (d18:1/23:1), SulfoHex (d18:2/23:0), and SulfoHex (t18:1/24:1) were significantly elevated, whereas hydroxylated or short-chain species such as SulfoHex (d18:0/18:0) and SulfoHex (d18:1/16:0) were markedly decreased. This remodelling pattern is consistent with findings in the MPTP-induced PD monkey model [65]. As critical components of myelin, STs play essential roles in maintaining membrane microdomain stability, glial cell function, and neuroinflammation [66, 67]. Accordingly, the observed reduction in specific hydroxylated STs may impair myelin integrity and glial homeostasis, thereby contributing to inflammation-associated neurodegeneration in PD. Arylsulfatase A (ASA), the lysosomal enzyme essential for ST catabolism, has been shown to exhibit decreased activity in patients with PD [68]. Such enzymatic deficiency may result in aberrant ST accumulation and lysosomal dysfunction, thereby contributing to key pathological features of PD, including *α*-syn accumulation, oxidative stress, and mitochondrial impairment. Moreover, alterations in ASA activity have been associated with early cognitive impairment and tremor-dominant PD phenotypes [69], supporting its potential utility as a biomarker for this disease.

**Fig. 10:**
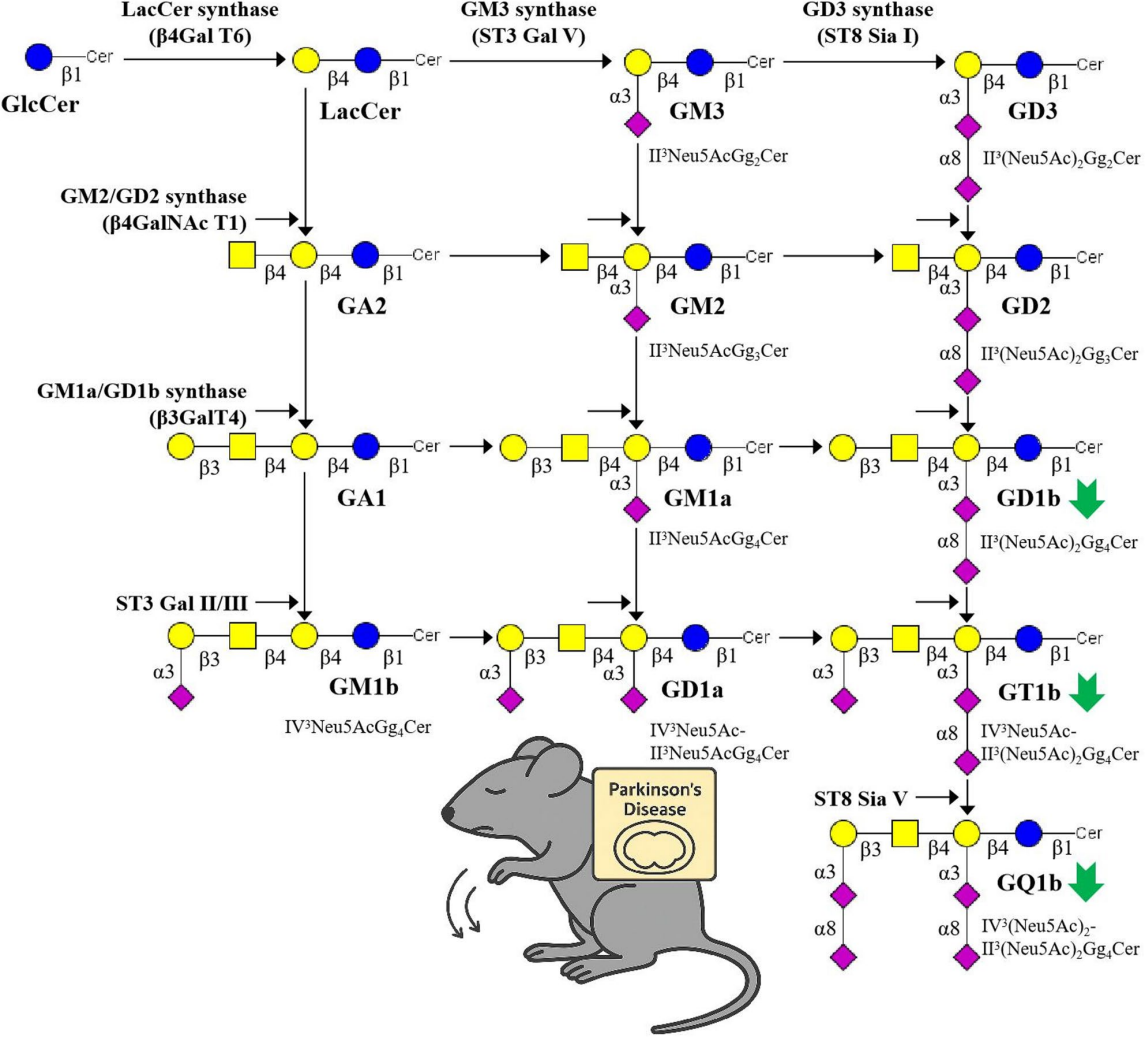
Proposed dysregulation of the GA biosynthetic pathway in PD mice. The green arrows indicate downregulated compounds

In addition, alterations in the synthesis pathway of GAs were analyzed. In this model, the findings suggest that the b-series GA synthesis pathway may be involved in the alterations observed in the brain tissues of PD mice. As shown in Fig. 10, the decrease in the GD1b/GD2 ratio suggested a blockage in the conversion of GD2 to GD1b. This step is primarily catalysed by *β*1,3-galactosyltransferase 4 (*B3galt4*), and its downregulation may account for the reduced levels of GD1b. Further analysis revealed a downward trend in the GT1b/GD1b and GQ1b/GT1b ratios, which may be associated with altered expression of sialyltransferase genes such as *St3gal2* and *St8sia5*. These observations suggest a possible link between polysialyltransferase activity and the imbalance in b-series GA synthesis in PD [70], although their regulatory roles require further experimental validation.

The obstruction of these biosynthetic pathways may alter the GA composition, affecting the stability of neuronal membranes and their ability to maintain homeostasis under stress, thereby increasing the risk of neurodegeneration [71]. Overall, *B3galt4*, *St3gal2*, and *St8sia5* may be involved in GA metabolism disorders in PD; subsequent studies could validate their expression via western blotting or qPCR to confirm their involvement in PD-associated ganglioside dysregulation.

## Strengths and limitations

In this study, the structural elucidation of acidic GSLs, particularly low-abundance isobaric or isomeric species, was significantly improved by adopting a prefractionation procedure combined with UHPLC-Q-TOF MS. Additionally, QSRR models were constructed to predict RTs and provide complementary information. This integrated strategy enabled a more comprehensive and accurate characterization of the acidic GSL profile in mouse brain tissue, addressing challenges posed by structural complexity and isomeric interference. The dMRM method markedly enhanced chromatographic peak resolution and signal intensity, particularly for low-abundance species or analytes with low ionization efficiency. This contributed to improved detection sensitivity and quantification precision, enabling more accurate and extensive GSL quantification in PD mouse brain tissues, and significantly enhancing the coverage of low-abundance GSLs.

The limitations of this study are mainly as follows. The biological sample size in this study is relatively limited, and the analysis was based on a single PD mouse model. Therefore, to enhance the generalizability and translational significance of the findings, future studies should validate these results in larger independent cohorts, different PD models, and clinical samples such as cerebrospinal fluid or plasma. Secondly, potential GSL biomarkers for PD were identified in this study; however, their clinical relevance will need further validation through clinical studies. In addition, the inferred disruptions in the b-series GA biosynthesis pathway should be further investigated through the expression and activity measurements of related sialyltransferases.

## Conclusion

Acidic GSLs in mouse brain tissues were comprehensively characterized. To the best of the authors’ knowledge, this study revealed the greatest number of acidic GSLs in mouse brain reported to date. Validated QSRR models were established, providing a supplementary tool that allowed the assisted identification of low-abundance species on the basis of retention time prediction. Moreover, this study achieved the most extensive quantitative coverage of acidic GSLs in the brains of PD model mice. Significant downregulation of GQ1b was reported as a potential PD-associated biomarker for the first time. If stable detection of these GSLs can be validated in cerebrospinal fluid or plasma from clinical patients, it may facilitate early diagnosis and more accurate stratification of PD. Unique GA modifications, such as O-acetylation, were also characterized, suggesting potential relevance to neural processes and disease progression. Additionally, disruptions in the b-series pathway may uncover potential therapeutic targets, with further investigation of key enzymes involved in this pathway likely guiding future PD treatment strategies. Collectively, this analysis illuminates the structural heterogeneity of acidic GSLs in mouse brain and underscores their diagnostic and therapeutic potential in PD.

## Supplementary Information

Supplementary Material 1. Figure. S1. Accumulation of α-synuclein (α-syn) in the substantia nigra pars compacta (SNpc) of A30P mice. (a) Representative immunofluorescence images of α-syn expression in the SNpc of A30P and Wild-Type mice. Scale bar = 5 μm. (b) Quantification of relative fluorescence intensity. Statistical significance was determined using an unpaired t-test. *, *P* < 0.05; **, *P* < 0.01; ***, *P* < 0.001.

Supplementary Material 2. Figure. S2. Loss of dopaminergic neurons in the substantia nigra pars compacta (SNpc) of A30P mice. (a) Representative immunohistochemical images of tyrosine hydroxylase (TH)-positive dopaminergic neurons in the SNpc of A30P and Wild-Type mice. Upper panels (scale bar = 200 μm), lower panels (scale bar = 50 μm). (b) Quantification of TH-positive neuron numbers in the SNpc. Statistical significance was determined using an unpaired t-test. *, *P* < 0.05; **, *P* < 0.01; ***, *P* < 0.001.

Supplementary Material 3. Figure. S3. Impaired motor coordination in A30P mice as assessed by rotarod test. Statistical significance was determined using an unpaired t-test. *, *P* < 0.05; **, *P* < 0.01; ***, *P* < 0.001.

Supplementary Material 4. Figure. S4. MS/MS spectrum of GM1a (d18:0/18:0)(OH) (Cpd.4).

Supplementary Material 5. Figure. S5. MS/MS spectrum of O-Ac-GM4 (d18:1/21:0) (Cpd.50).

Supplementary Material 6. Figure. S6. MS/MS spectrum of GQ1b (d20:1/23:0) (Cpd.159).

Supplementary Material 7. Figure. S7. MS/MS spectrum of O-Ac-GQ1b (d18:0/20:0) (Cpd.160).

Supplementary Material 8. Figure. S8. MS/MS spectrum of O-Ac-GQ1b (d18:1/23:0) (Cpd.163).

Supplementary Material 9. Figure. S9. MS/MS spectrum of O-Ac-GQ1b (d20:1/22:0) (Cpd.168).

Supplementary Material 10. Figure. S10. MS/MS spectrum of GP1c (d20:1/20:0) (Cpd.173).

Supplementary Material 11. Figure. S11. MS/MS spectrum of SulfoHex (t18:1/21:1) (Cpd.213).

Supplementary Material 12. Figure. S12. Intra-day and inter-day precision assay of QC samples.

Supplementary Material 13. Figure. S13. OPLS-DA score plot of brain tissues samples of mice in PD group (red, *n* = 7) and HC group (green, *n* = 5) (a); VIP scores plot (b); permutations plot (*R*^2^Y = 0.951, Q^2^ = 0.767) (c).

Supplementary Material 14.

## Data Availability

The datasets used and analyzed during the current study are available from the corresponding author on reasonable request.

## Abbreviations

GSLs: Glycosphingolipids
GAs: Gangliosides
STs: Sulfatides
QSRR: Quantitative structure–retention relationship
dMRM: Dynamic multiple reaction monitoring
SA: Sialic acid
PD: Parkinson’s disease
CNS: Central nervous system
UHPLC: Ultra-high-performance liquid chromatography
GM: Monosialoganglioside
GD: Disialoganglioside
GT: Trisialoganglioside
GQ: Tetrasialoganglioside
GP: Pentasialoganglioside
MS: Mass spectrometry
Q-TOF: Quadrupole time-of-flight
LC: Liquid chromatography
IM: Ion mobility
RT: Retention time
QQQ: Triple quadrupole
MRM: Multiple reaction monitoring
α-syn: α-synuclein
CE: Collision energy
QC: Quality control sample
LOD: Limit of detection
IS: Internal standard
LOQ: Limit of quantitation
S/N: Signal-to-noise ratio
FBF: Find by formula
GA-MLR: Genetic algorithm–multiple linear regression
RMSE: Root mean square error
LOO: Leave-one-out
OPLS-DA: Orthogonal partial least squares discriminant analysis
VIP: Variable importance in projection
AD: Alzheimer’s disease
RSD: Relative standard deviation
HC: Healthy control
