# Supplementary figures and images for: Exploration of the structural diversity and distribution pattern of gangliosides and sulfatides in mouse brain tissues and biomarkers for Parkinson’s Disease
Source: Lipids Health Dis. 2026 Mar 25;25:123. doi: 10.1186/s12944-026-02918-1 (PMC13137580; doi:10.1186/s12944-026-02918-1)

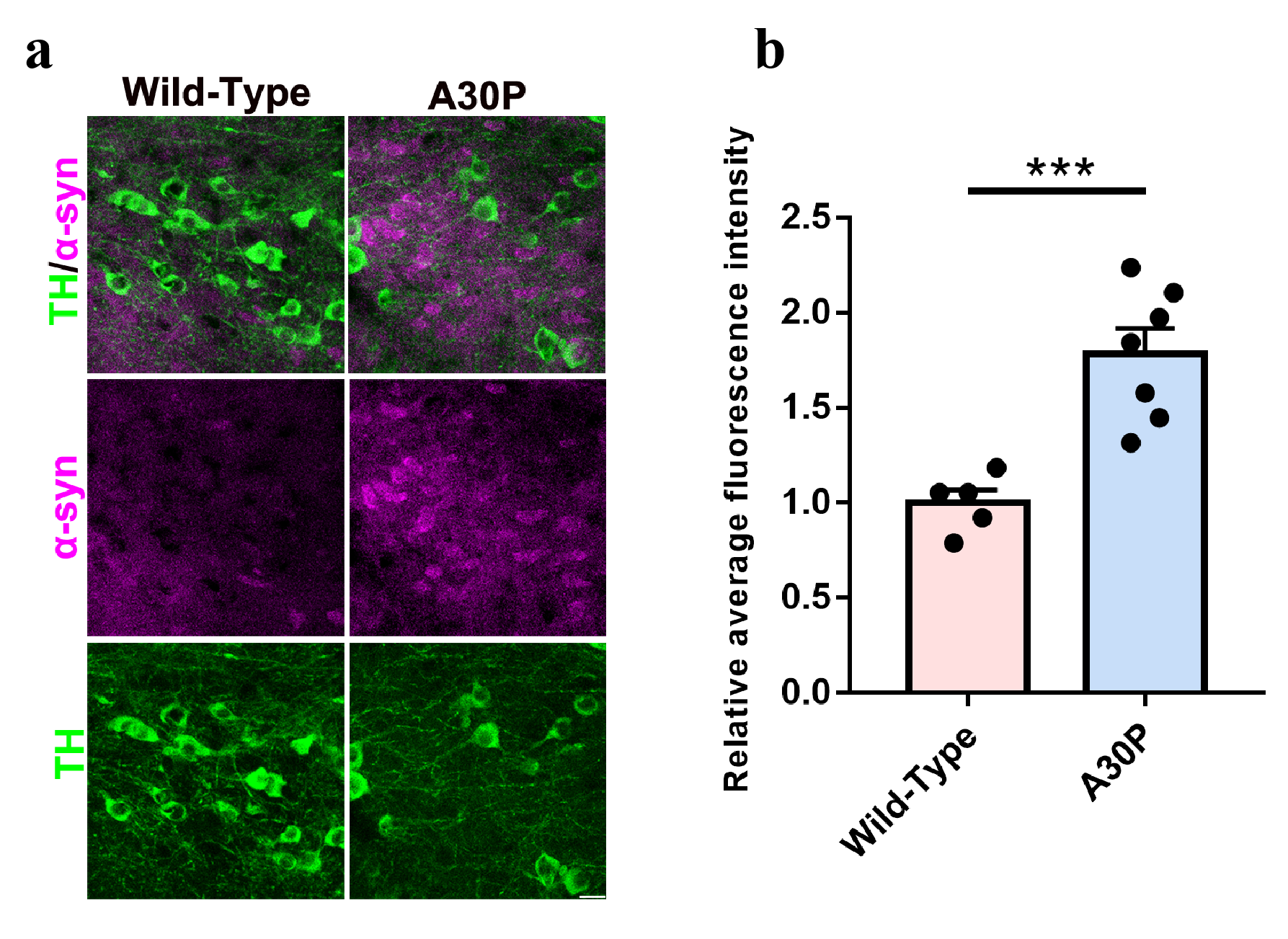

Supplement: Supplementary file 1 — Supplementary Material 1. Figure. S1. Accumulation of α-synuclein (α-syn) in the substantia nigra pars compacta (SNpc) of A30P mice. (a) Representative immunofluorescence images of α-syn expression in the SNpc of A30P and Wild-Type mice. Scale bar = 5 μm. (b) Quantification of relative fluorescence intensity. Statistical significance was determined using an unpaired t-test. *, P < 0.05; **, P < 0.01; ***, P < 0.001. [file 12944_2026_2918_MOESM1_ESM.tif]

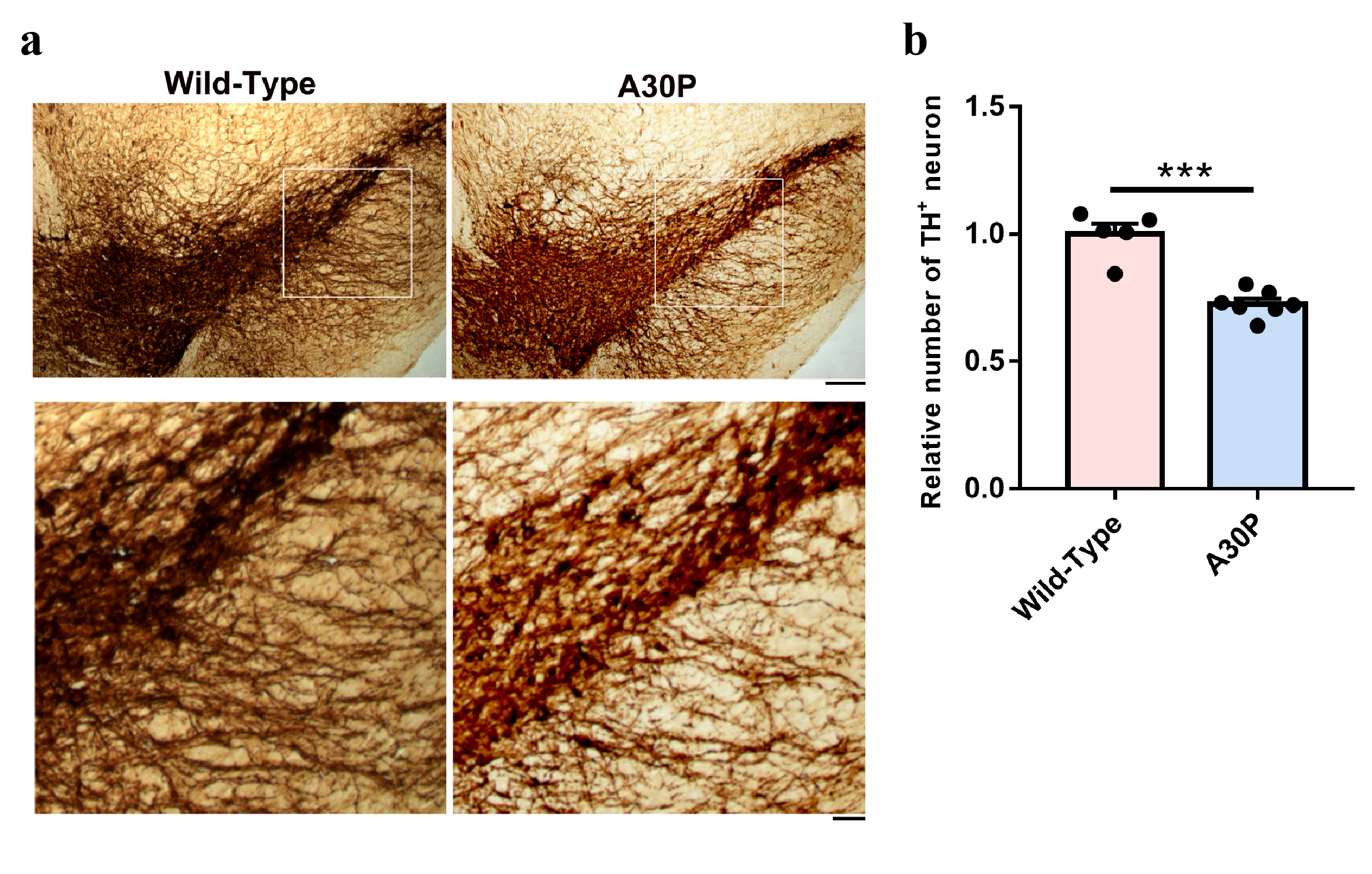

Supplement: Supplementary file 2 — Supplementary Material 2. Figure. S2. Loss of dopaminergic neurons in the substantia nigra pars compacta (SNpc) of A30P mice. (a) Representative immunohistochemical images of tyrosine hydroxylase (TH)-positive dopaminergic neurons in the SNpc of A30P and Wild-Type mice. Upper panels (scale bar = 200 μm), lower panels (scale bar = 50 μm). (b) Quantification of TH-positive neuron numbers in the SNpc. Statistical significance was determined using an unpaired t-test. *, P < 0.05; **, P < 0.01; ***, P < 0.001. [file 12944_2026_2918_MOESM2_ESM.tif]

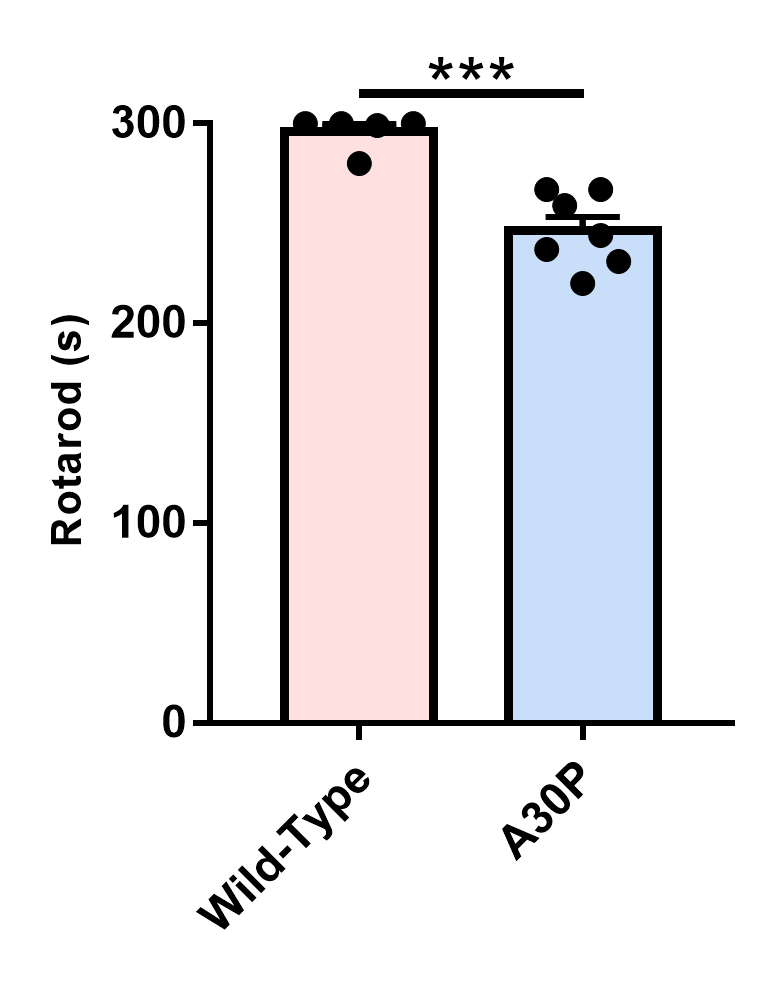

Supplement: Supplementary file 3 — Supplementary Material 3. Figure. S3. Impaired motor coordination in A30P mice as assessed by rotarod test. Statistical significance was determined using an unpaired t-test. *, P < 0.05; **, P < 0.01; ***, P < 0.001. [file 12944_2026_2918_MOESM3_ESM.tif]

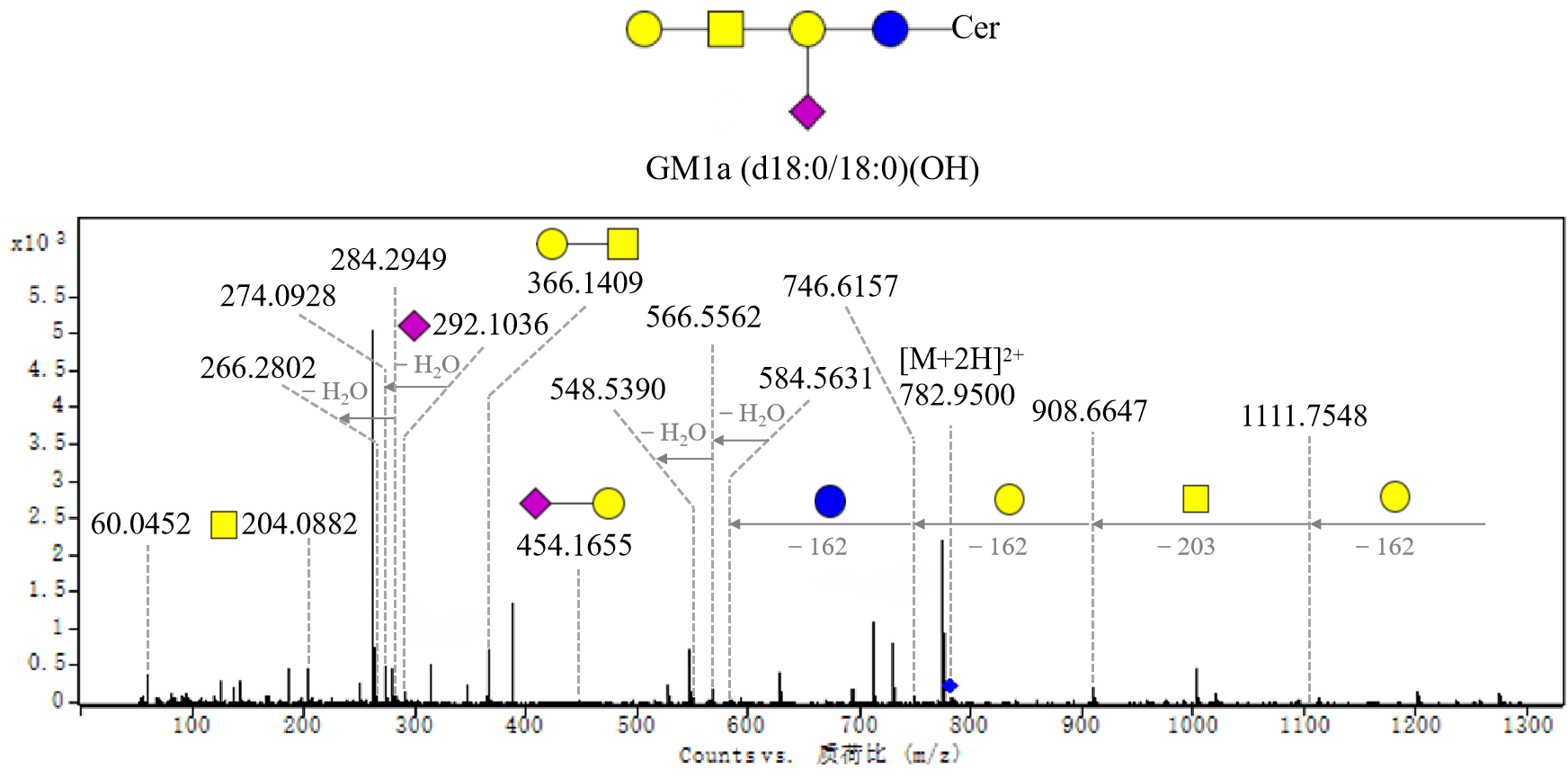

Supplement: Supplementary file 4 — Supplementary Material 4. Figure. S4. MS/MS spectrum of GM1a (d18:0/18:0)(OH) (Cpd.4). [file 12944_2026_2918_MOESM4_ESM.tif]

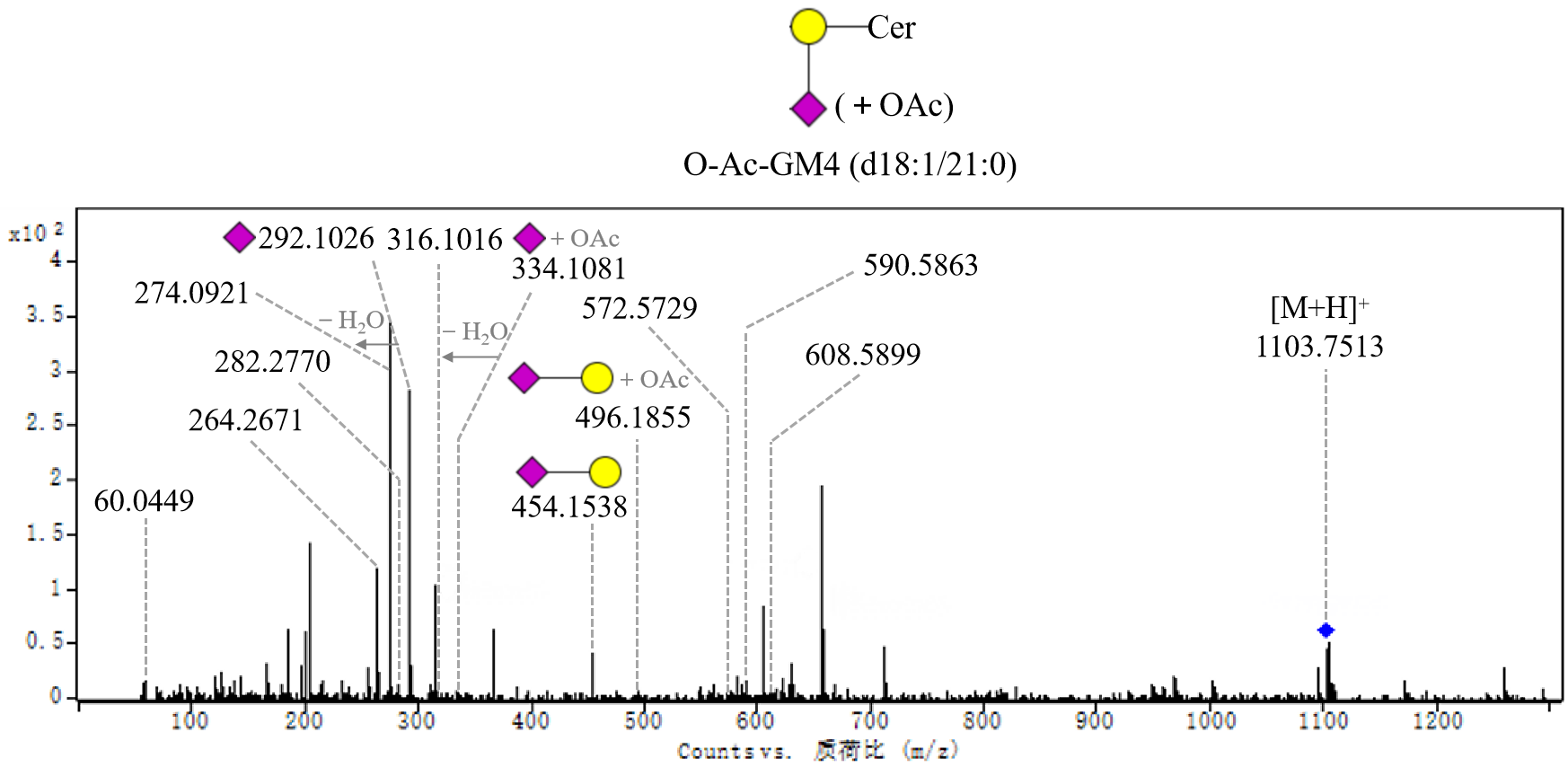

Supplement: Supplementary file 5 — Supplementary Material 5. Figure. S5. MS/MS spectrum of O-Ac-GM4 (d18:1/21:0) (Cpd.50). [file 12944_2026_2918_MOESM5_ESM.tif]

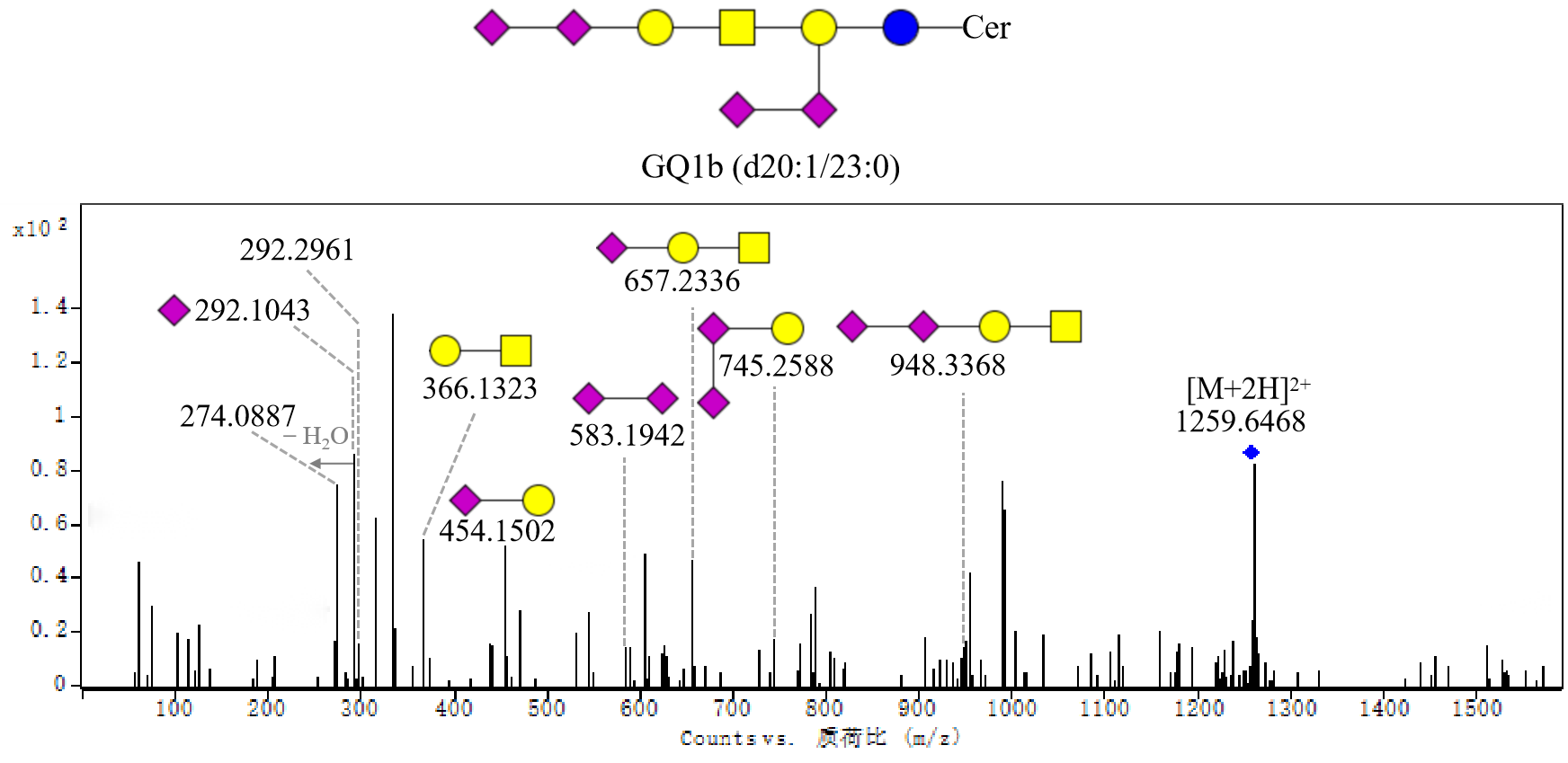

Supplement: Supplementary file 6 — Supplementary Material 6. Figure. S6. MS/MS spectrum of GQ1b (d20:1/23:0) (Cpd.159). [file 12944_2026_2918_MOESM6_ESM.tif]

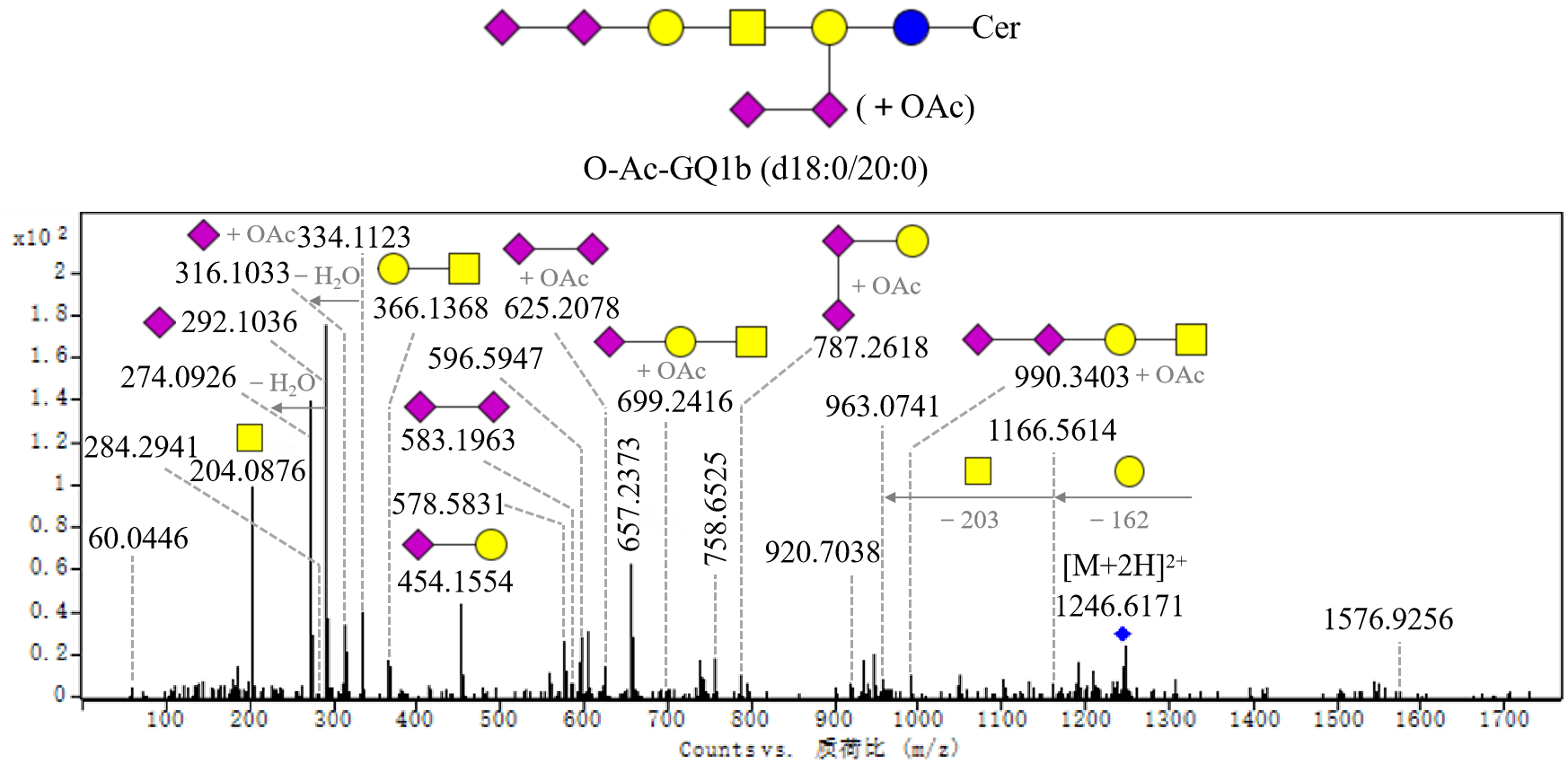

Supplement: Supplementary file 7 — Supplementary Material 7. Figure. S7. MS/MS spectrum of O-Ac-GQ1b (d18:0/20:0) (Cpd.160). [file 12944_2026_2918_MOESM7_ESM.tif]

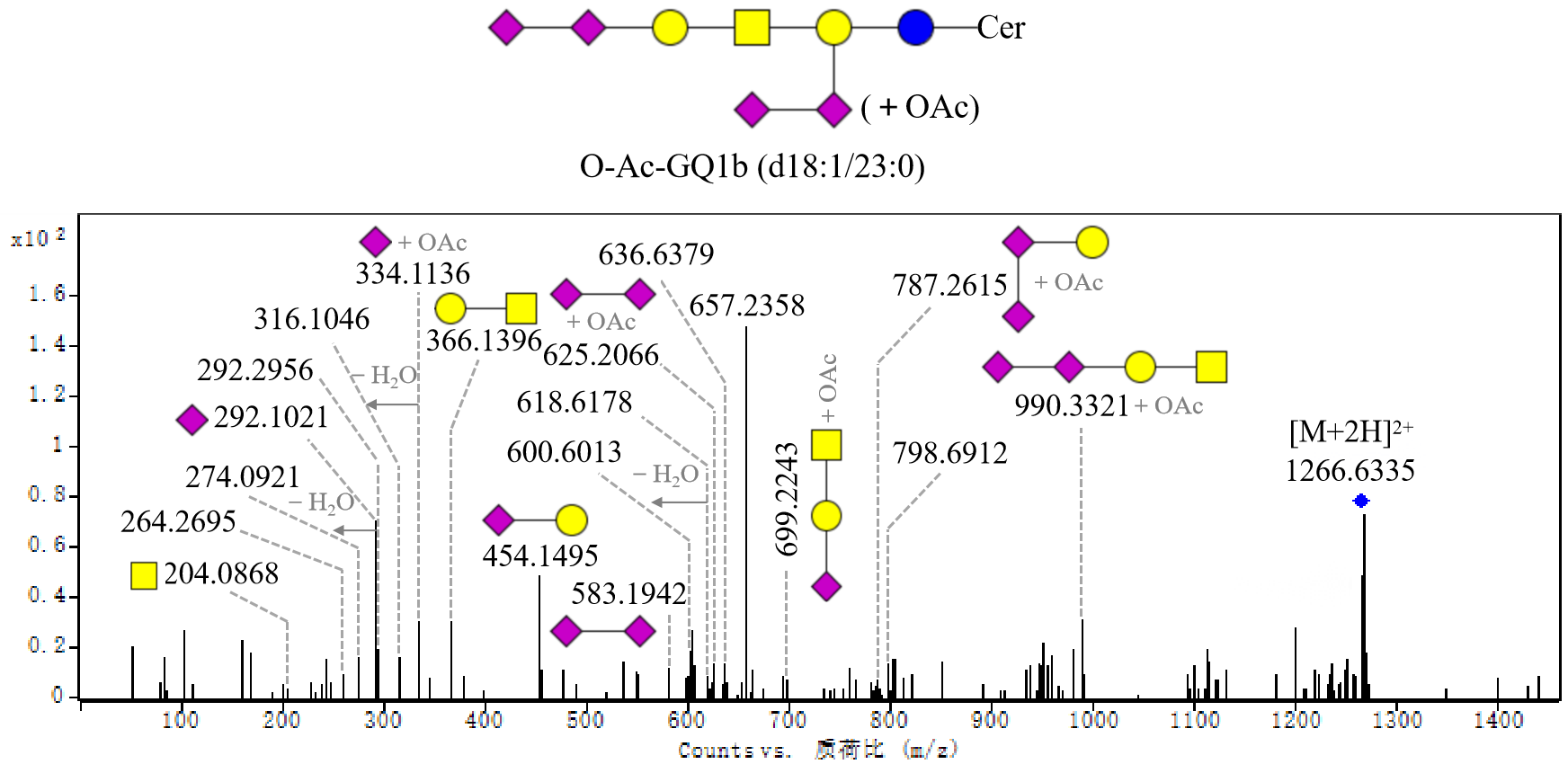

Supplement: Supplementary file 8 — Supplementary Material 8. Figure. S8. MS/MS spectrum of O-Ac-GQ1b (d18:1/23:0) (Cpd.163). [file 12944_2026_2918_MOESM8_ESM.tif]

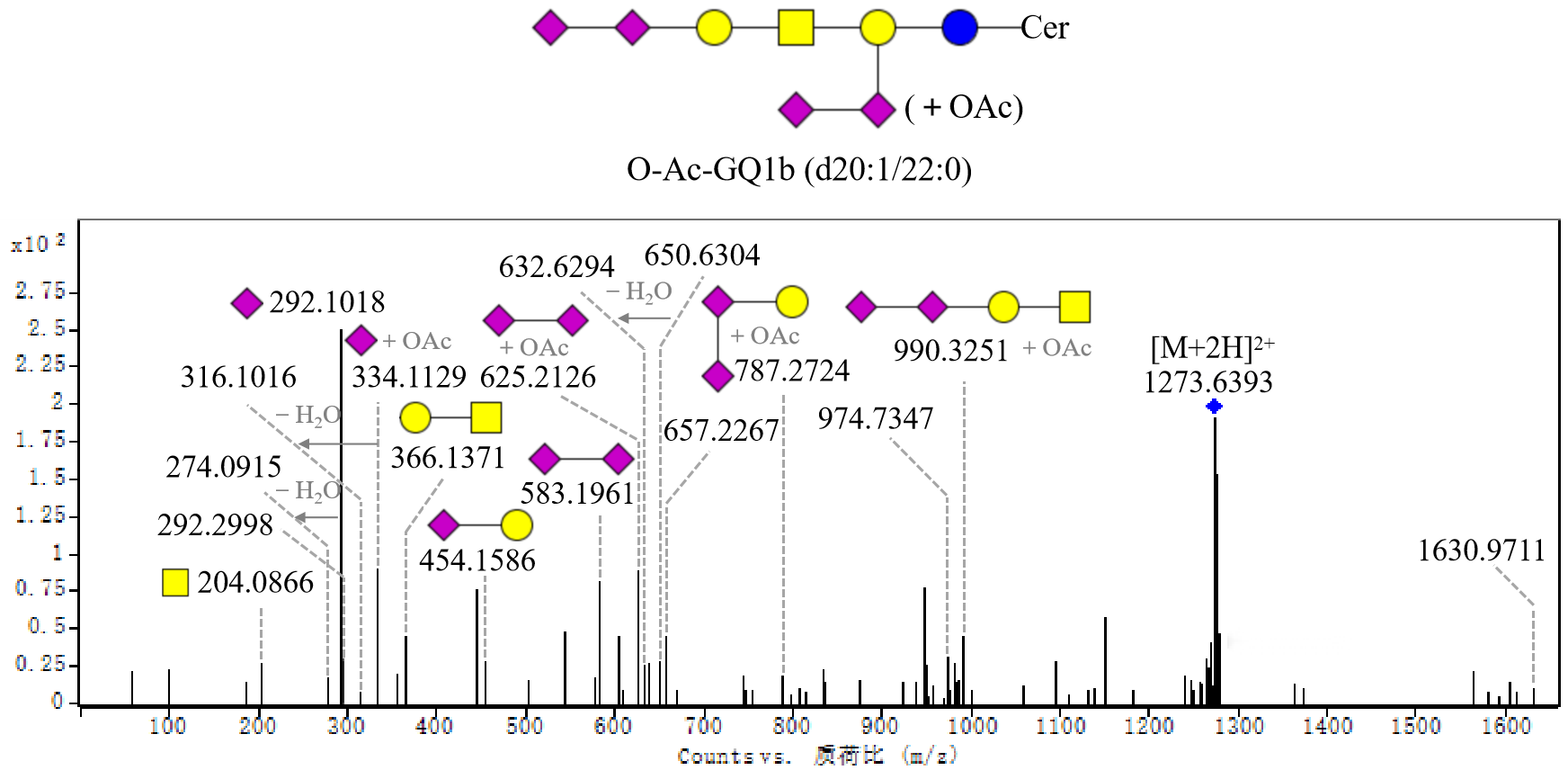

Supplement: Supplementary file 9 — Supplementary Material 9. Figure. S9. MS/MS spectrum of O-Ac-GQ1b (d20:1/22:0) (Cpd.168). [file 12944_2026_2918_MOESM9_ESM.tif]

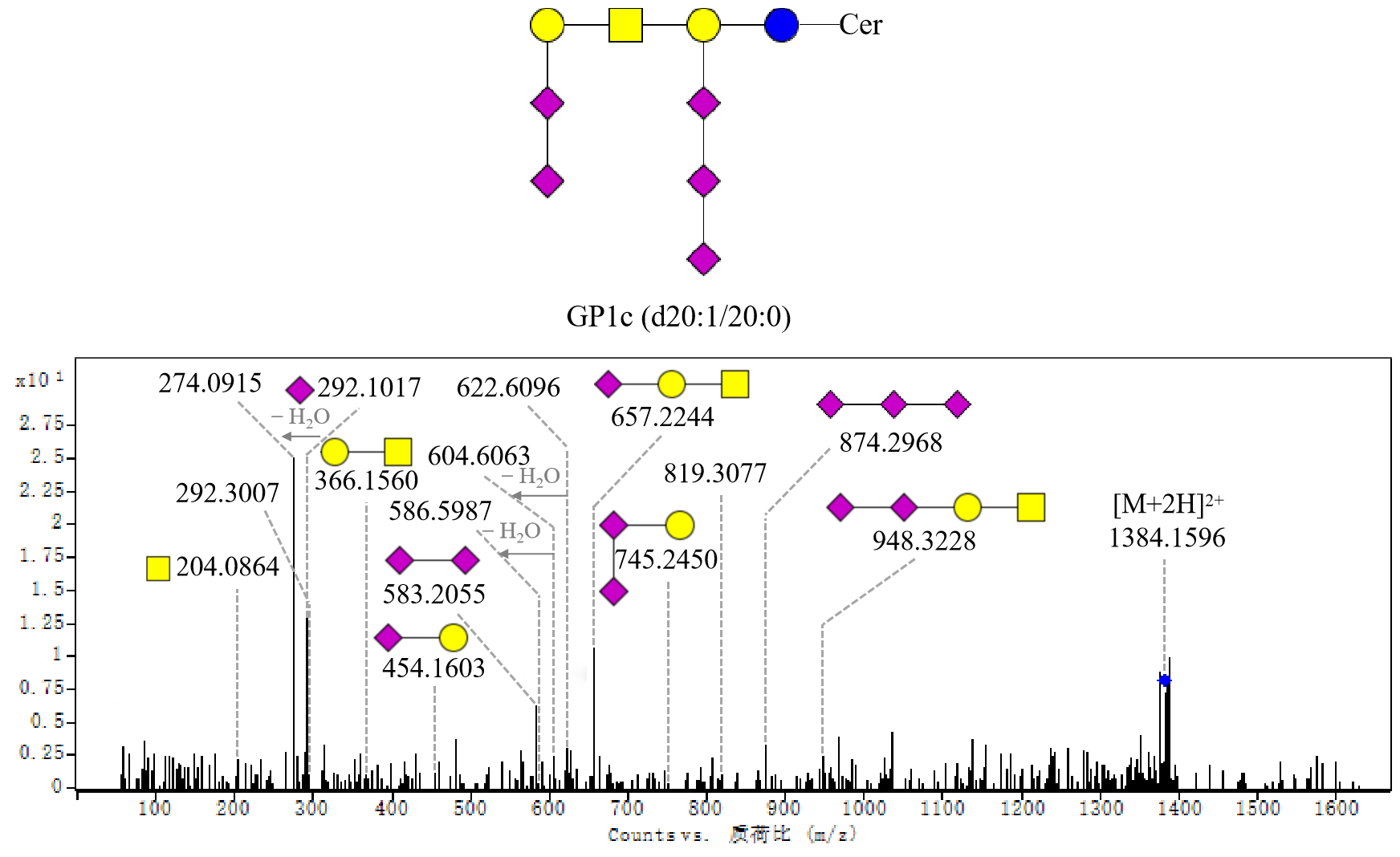

Supplement: Supplementary file 10 — Supplementary Material 10. Figure. S10. MS/MS spectrum of GP1c (d20:1/20:0) (Cpd.173). [file 12944_2026_2918_MOESM10_ESM.tif]

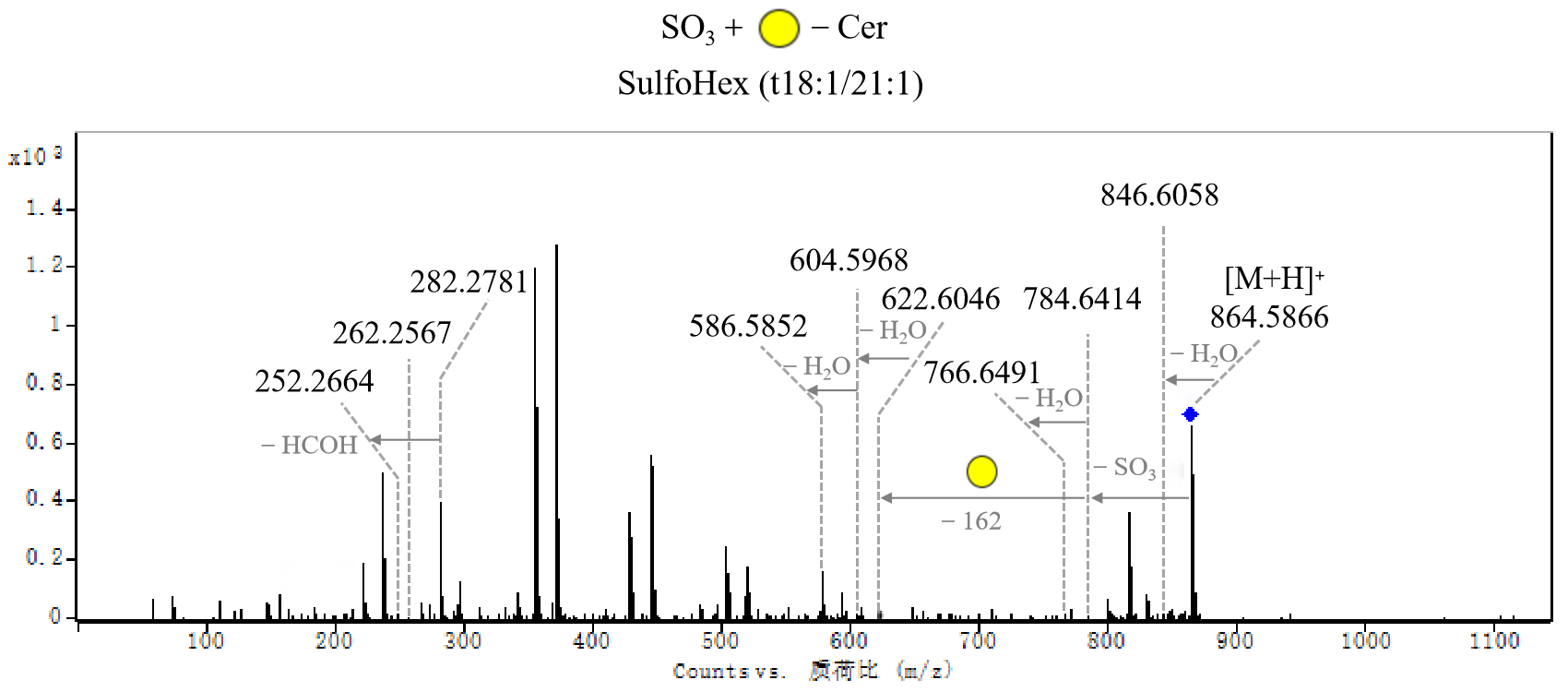

Supplement: Supplementary file 11 — Supplementary Material 11. Figure. S11. MS/MS spectrum of SulfoHex (t18:1/21:1) (Cpd.213). [file 12944_2026_2918_MOESM11_ESM.tif]

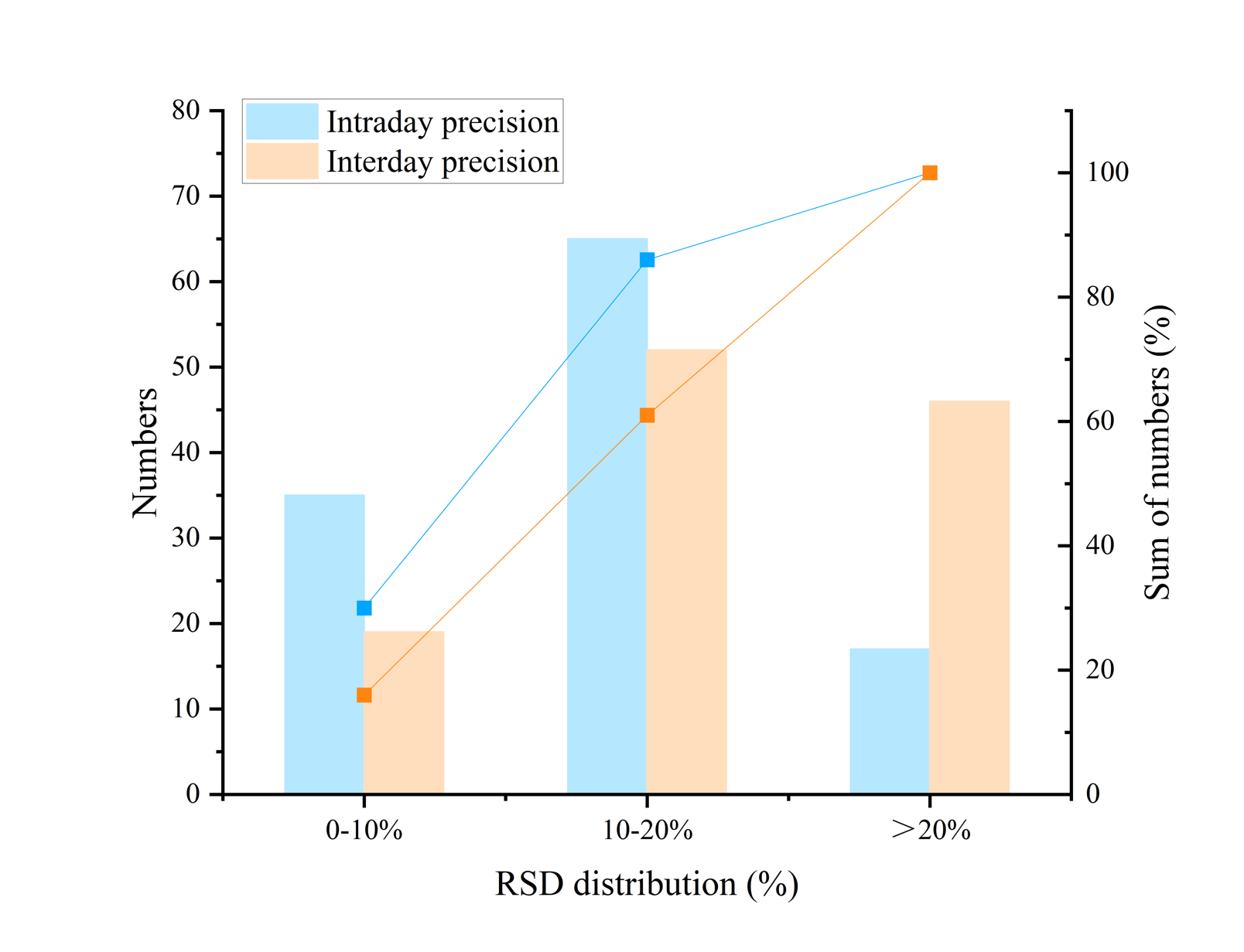

Supplement: Supplementary file 12 — Supplementary Material 12. Figure. S12. Intra-day and inter-day precision assay of QC samples. [file 12944_2026_2918_MOESM12_ESM.tif]

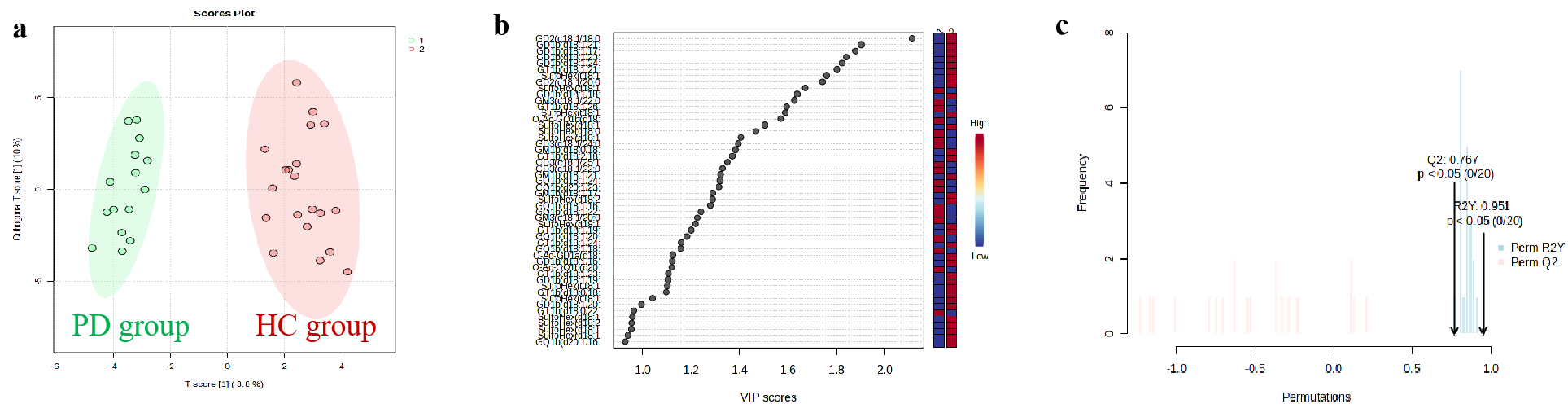

Supplement: Supplementary file 13 — Supplementary Material 13. Figure. S13. OPLS-DA score plot of brain tissues samples of mice in PD group (red, n = 7) and HC group (green, n = 5) (a); VIP scores plot (b); permutations plot (R2Y = 0.951, Q2 = 0.767) (c). [file 12944_2026_2918_MOESM13_ESM.tif]
